# A 3-dimensional mathematical model of microbial proliferation that generates the characteristic cumulative relative abundance distributions in gut microbiomes

**DOI:** 10.1371/journal.pone.0180863

**Published:** 2017-08-08

**Authors:** Lena Takayasu, Wataru Suda, Eiichiro Watanabe, Shinji Fukuda, Kageyasu Takanashi, Hiroshi Ohno, Misako Takayasu, Hideki Takayasu, Masahira Hattori

**Affiliations:** 1 Graduate School of Frontier Sciences, The University of Tokyo, 5-1-5, Kashiwanoha, Kashiwa, Chiba, 277-8561, Japan; 2 School of Medicine, Keio University, 35 Shinanomachi, Shinjuku-ku, Tokyo, 160-8582, Japan; 3 RIKEN Center for Integrative Medical Sciences, 1-7-22 Suehiro-cho, Tsurumi-ku, Yokohama City, Kanagawa, 230-0045, Japan; 4 Institute for Advanced Biosciences, Keio University, 246-2 Mizukami, Kakuganji, Tsuruoka, Yamagata, 997-0052, Japan; 5 Institute of Innovative Research, Tokyo Institute of Technology, G3-52, 4259, Nagatsutacho, Midori-ku, Yokohama-shi, Kanagawa 226-8502, Japan; 6 Sony Computer Science Laboratories, Inc. 3-14-13, Higashigotanda, Shinagawa-ku, Tokyo, 141-0022, Japan; 7 Graduate School of Advanced Science and Engineering, Waseda University, 3-4-1 Okubo Shinjuku-ku, Tokyo, 169-8555, Japan; Memorial Sloan-Kettering Cancer Center, UNITED STATES

## Abstract

The gut microbiome is highly variable among individuals, largely due to differences in host lifestyle and physiology. However, little is known about the underlying processes or rules that shape the complex microbial community. In this paper, we show that the cumulative relative abundance distribution (CRAD) of microbial species can be approximated by a power law function, and found that the power exponent of CRADs generated from 16S rRNA gene and metagenomic data for normal gut microbiomes of humans and mice was similar consistently with ∼0.9. A similarly robust power exponent was observed in CRADs of gut microbiomes during dietary interventions and several diseases. However, the power exponent was found to be ∼0.6 in CRADs from gut microbiomes characterized by lower species richness, such as those of human infants and the small intestine of mice. In addition, the CRAD of gut microbiomes of mice treated with antibiotics differed slightly from those of infants and the small intestines of mice. Based on these observations, in addition to data on the spatial distribution of microbes in the digestive tract, we developed a 3-dimensional mathematical model of microbial proliferation that reproduced the experimentally observed CRAD patterns. Our model indicated that the CRAD may be determined by the ratio of emerging to pre-existing species during non-uniform spatially competitive proliferation, independent of species composition.

## Introduction

The human gut harbors trillions of microbes belonging to hundreds of species. The structure of the gut microbiome is influenced by individual lifestyle, including dietary habits and host physiology, including disease, resulting in a high degree of interindividual variability in species richness and abundance [1–8]. In addition to many studies of the ecological and biological factors that influence the diversity of human gut microbiomes, several studies have reported the universal features shared among individual gut microbiomes. For instance, some studies found that human gut microbiomes can be classified into a few distinct clusters, called enterotypes, that are characterized by the relative abundance of a few particular taxa [9, 10]. Some groups of the gut species exhibit robust bistable abundance distributions [11]. Host-independent dynamics of the high interindividual variability in the human gut microbiome have also been observed [12]. Although these studies revealed several fundamental features of the structure of the gut microbiome, independent of the high variability, studies that explore the underlying rules and processes that shape the human gut microbiome remain scarce.

Metagenomic analysis of human gut microbiomes by high-throughput sequencing revealed long-tailed distributions of relative species abundance in the microbial community structure [13, 14]. Such long-tailed distributions have also been observed in various natural ecosystems, such as coral reefs and rainforests, soil microbial communities, and marine phage communities [15–18]. Recently, several studies attempted to explain the species abundance distribution by fitting some known mathematical models for ecosystem including the neutral theory which assumes an equal opportunity for all microbes to birth, dying, migrating, and speciation for all microbes. However, these models still have some discrepancy with the observed data, and thus further improvements are necessary for precisely deducing the process shaping the ecosystem and community structures [19–21]. In this study, we investigate the cumulative relative abundance distribution (CRAD) of species in gut microbiomes and reveal that it follows power law; the power exponent was consistent in both normal and altered gut microbiomes of humans and mice. In addition, we observed different CRAD patterns in several gut microbiomes, including those of human infants and the small intestines of mice. Based on these experimentally obtained CRADs, we developed a mathematical model of microbial proliferation, and demonstrated that the CRAD might be generated under spatially uneven competitive proliferation of microbes in the digestive tract.

## Materials and methods

### Ethics statement

This study was approved by the Research Ethics Committee of the University of Tokyo and the Human Research Ethics Committee of Azabu University, and written informed consent was obtained from all the subjects. All animal experiments were approved by the Animal Research Committee of RIKEN Yokohama Institute.

### Dissection of mouse gut and DNA extraction

The whole intestine of six specific pathogen-free (SPF) mice was dissected into six sections (S6A Fig). The contents were washed out using l mL of PBS buffer and subjected to the following DNA extraction process. The collected contents were pelleted by centrifugation and then the supernatant was discard. The pellet was suspended in 200 *μ*L of 10% sodium dodecyl sulfate/1×TE buffer. Proteinase K solution (Merck) was then added and the sample was incubated at 37°C for 1 h. The samples were mixed with 0.1 g zirconia beads, 200 *μ*L of 3 M sodium acetate, 400 *μ*L of phenol/chloroform/isoamyl alcohol. The sample was vigorously mixed in a Shake Master Mini, ver.16 (BMS) at 1,500 rpm for 3 min. The sample was then centrifuged at 13,000 g at 4°C for 10 min. The supernatant was transferred to a new tube and mixed with 400 *μ*L of phenol/chloroform/isoamyl alcohol. The tubes were centrifuged at 13,000 g at 4°C for 10 min and the supernatant was transferred to a new tube, and the DNA was precipitated by adding 3 M sodium acetate (1/10 volume of the DNA solution) and two volumes of ethanol to the supernatant. DNA was pelleted by centrifugation at 13,000 g at 4°C for 15 min. The DNA pellet was rinsed with 75% ethanol, vacuum dried, and dissolved in TE buffer.

### Meta 16S sequencing

The hypervariable V1-V2 regions of the 16S rRNA gene were amplified by PCR using universal primers (a barcoded 27Fmod and 338R), and multiplexed amplicon pyrosequencing was carried out using a 454 GS FLX Titanium or 454 GS JUNIOR sequencer (Roche Applied Science), as previously described [22]. The 16S rRNA sequence data generated in this study were deposited in DDBJ/GenBank/EMBL under accession numbers DRA005152, DRA000869-DRA000886, DRA002875-DRA002906, DRA002611, DRA002617, and DRA002618.

### Processing of 16S sequence data

The 16S sequences were assigned to samples on the basis of their barcode sequence. Reads with an average quality value <25 and those lacking the primer sequences at both ends were excluded. Possibly chimeric sequences that had alignment lengths of <90% coverage with authentic reference 16S sequences in the database (described below) were also removed. The filtered reads obtained from these quality-control processes accounted for approximately 50% of the total reads. The majority of the excluded reads lacked PCR primer sequences. After trimming both primer sequences from the filtered reads, 2,500 reads per sample were randomly selected and used for further analyses. The high-quality reads were sorted according to quality value and then clustered into operational taxonomic units (OTUs) using a 96% pairwise-identity cutoff with the UCLUST program [23] v.5.2.32 (http://www.drive5.com/). Representative sequences of the generated OTUs were BLAST searched against our 16S database constructed for this study, using the GLSEARCH program to determine the closest taxa. The 16S database was constructed from publicly available databases: Ribosomal Database Project (RDP) v.10.31, CORE (http://microbiome.osu.edu/) and a reference genome sequence database obtained from the NCBI FTP site (ftp://ftp.ncbi.nih.gov/genbank/, December 2011). For assignment at the genus level, a sequence similarity threshold of 94% was applied.

### Cumulative relative abundance distribution (CRAD)

Relative abundance distribution (RAD) is a known analytic method for visualizing the frequency of observed species; it is typically fitted by power law. The exponent serves as an index of the biodiversity of the ecosystem. The figures show cumulative distribution plots of relative abundance, which is equivalent to the rank-frequency plot, by exchanging the vertical and horizontal axes, as the rank is given by the product of the total number of species and the cumulative distribution value. For the 16S analysis results, the X-axis indicates the number of OTUs in the 2,500 reads per sample. Y is given by

$$\begin{array}{c} Y=F_{X}\left(x\right)=P\left(X\leq x\right) \end{array}$$ (1)

In the metagenomic analysis, the X-axis represents the pseudo-read number, which is obtained from the 2,500 random metagenomic data samples, based on a probability proportional to the abundance data. The Y-axis represents the same as above. The average CRAD was calculated by splitting the CRADs from multiple samples into logarithmic bins; the median value for each bin was used as the representative value. We calculated *β* value of the abundance range from 0.8 to 40% by the MLE, in which the samples with few data points (<2) in the range and those with the *p*-values <0.1 in the Goodness-of-fit test were removed from the calculation.

### Histological analysis

The colon and distal small intestine of the mice were fixed in methacarn solution and embedded in paraffin. For mucin visualization, sections were stained with rabbit anti-Muc2 antibodies (Santa Cruz Biotechnology) followed by Alexa 488-labeled goat anti-rabbit IgG (Life Technologies). For fluorescence in situ hybridization staining (FISH) of bacterial strains, the sections were hybridized overnight with 5’ Alexa 555-labeled EUB 338 probes. All sections were counterstained with DAPI, mounted with Fluoromount/Plus (Diagnostics BioSystems) and visualized using a Leica TCS SP5 confocal microscope.

### Mathematical model

We assumed a stochastic evolution model in a three-dimensional lattice (*x*, *y*, *z*). That is, a site is either empty or occupied by a species of bacteria. Initially, all sites are empty and bacteria are located only in the bottom layer (*z* = 0) of 40 × 40 sites, which is considered to be the bacterial assemblage surface of the intestine. Bacteria are assumed to grow randomly to a neighboring vacant site, and new bacterial species immigrate with a constant probability of 1-*p*. For numerical simulation, the competition process is performed by repeating the following operations 40 × 40 × 2000 times. (1) Select a vacant site in each layer successively, chosen in order from bottom to top. (2) Randomly choose an occupied neighboring site in the lower layer, with a probability *p*, or let a new bacterial species immigrate into the site, with a probability 1-*p*, where the periodic boundary condition in the *x*-y direction is applied for the boundary sites. (3) Let the selected neighboring bacteria or new species of bacteria grow into the chosen vacant site. By repeating the operations above, the bacterial species that have already grown will have an increasing chance of growth, following the “rich get richer” rule. Species that grow a little faster than other species are likely to be selected again. That is why this simple rule reproduces a power law distribution, even from indiscriminate conditions. When we defined the growth rate of each species, we attributed non-uniform weights to the probability of being selected depending on the species.

## Results and discussion

### Robustness of CRAD in gut microbial community

#### CRADs of microbiome in Japanese adults

**Fig 1:**
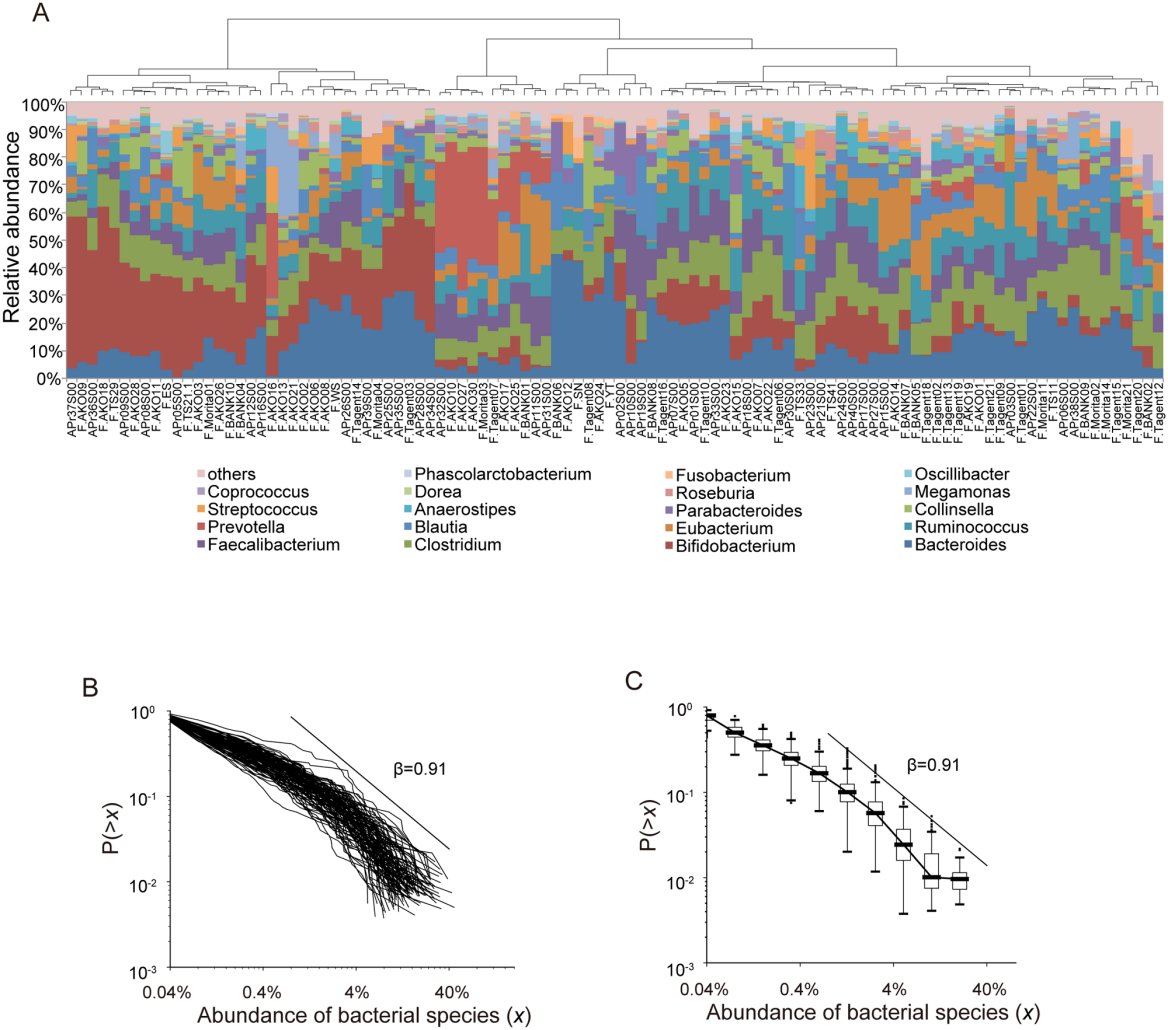
CRADs of human gut microbiomes. A: Clustering of the 104 healthy Japanese samples. Hierarchal clustering is performed at the genus-level abundance of the 104 Japanese samples based on Bray-Curtis distances. B: Individual CRADs of the 104 healthy Japanese gut microbiomes based on the OTU-level composition. C: Median CRADs of the same samples as those in (B).

We obtained 16S ribosomal RNA gene (16S) V1-V2 sequences from fecal samples collected from healthy Japanese adults [24]. Clustering analysis of the 16S sequences showed that the samples were roughly segregated to several groups, indicating the high inter-individual variability in the human gut microbiomes as reported previously [14] (Fig 1A). We calculated the CRADs using the OTU-level abundance of each subject, and found that the CRADs showed a linear relationship in log-log plots that was similar between the all subjects (Fig 1B). In addition, these CRADs showed a long-tailed structure that could be well approximated by a power law function with a power exponent (*β*) of ∼0.9 estimated by the maximum likelihood estimation (MLE) in the range between 0.8 and 40% in abundance (Fig 1C and S1 Table). To further validate that the CRAD follows power law, we compared the fitness of power law to the CRADs with that of several other heavy tailed distributions such as log-normal and stretched exponential distributions (S1 Fig) [25]. The results for the analysis using the 104 Japanese data showed that *p* values (the Goodness-of-fit test) were 0.489, 0.010, and 0.001 for power law, log-normal, and stretched exponential, respectively, indicating that only power law significantly fit to the CRAD, while log-normal and stretched exponential were rejected according to the Newman’s standard in which the distribution hypothesis is plausible when *p*-value is greater than 0.1. Similar *β* values of CRAD (0.88 in average) were also observed in the analysis of the metagenomic dataset from the same Japanese samples (S1 Table and S2 Fig) [24]. These results suggest the existence of a hitherto unknown rule that generates characteristic CRADs independent of species composition which is affected by different lifestyles including diet and genetic backgrounds of individuals. The power law observed in CRADs motivated our search for the mechanisms underlying the gut microbial community structure.

#### CRADs of murine gut microbiome

**Fig 2:**
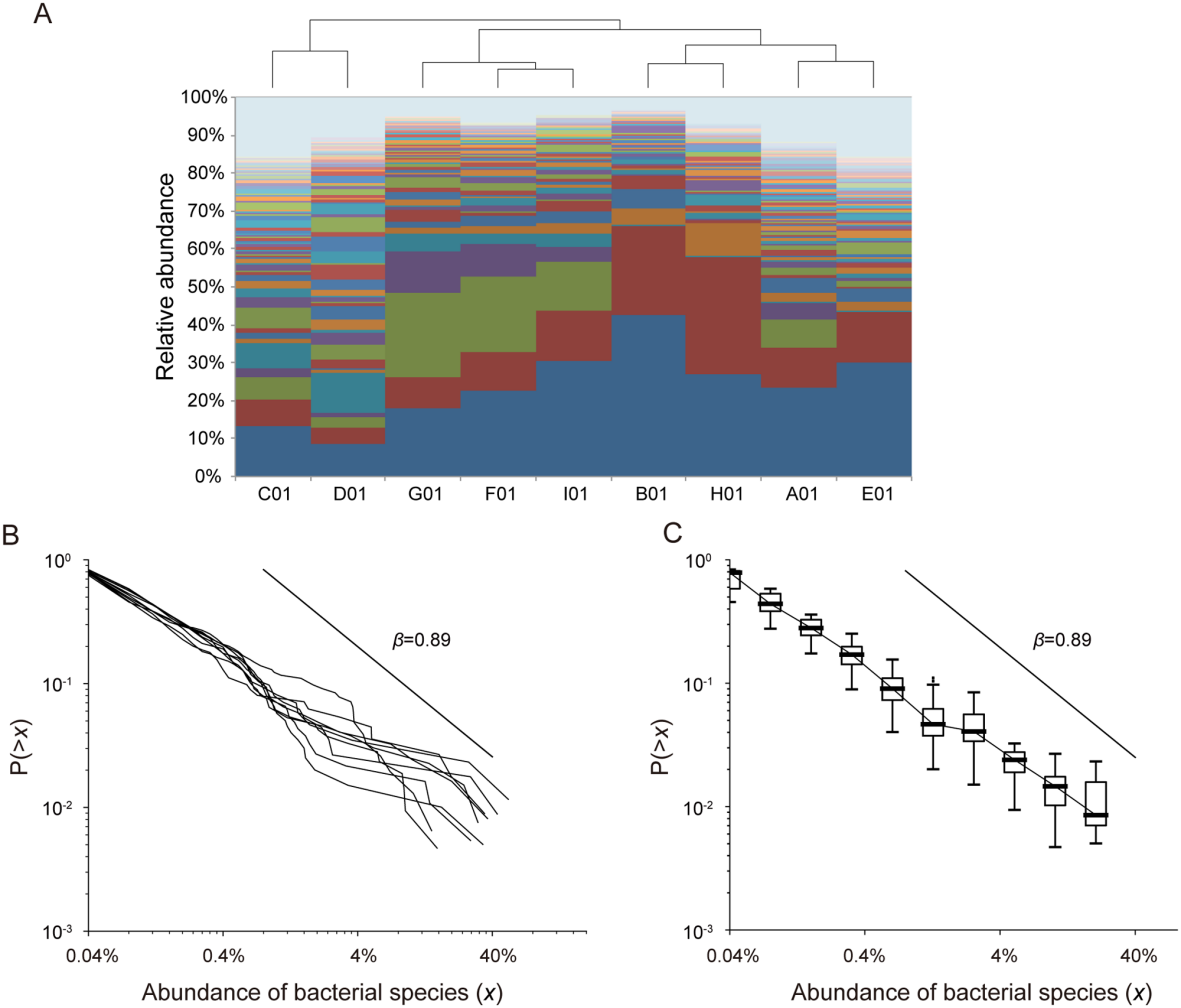
CRADs of murine gut microbiomes. A: Clustering of the bacterial composition at the OTU level in mice. Hierarchal clustering is performed at the OTU-level abundance of the samples. Each OTU is shown in a different color. B: Individual CRADs of murine gut microbiomes based on the OTU-level composition. C: Median CRADs of the same samples as those in (B).

We next determined the CRAD of murine gut microbiomes. We collected fecal samples from conventional mice and obtained the 16S V1-V2 sequences of the microbiome from them. Although the OTU-level abundance was varied among the nine mice (Fig 2A), all of the gut microbiomes had a characteristic CRAD that could be approximated by a power law function with a *β* of 0.89 (Fig 2B and 2C, and S1 Table), similar to that observed in the human adult dataset described above. We tested the similarity of *β* values between the samples of humans and mice, and the results revealed that the both *β* values estimated by the MLE were not significantly different by ANOVA assessment (P = 0.64, S2 Table). These results also suggested the existence of a common rule generating similar CRAD patterns in the gut microbiomes of both humans and mice.

#### CRADs of human gut microbiomes with dietary intervention

To examine the effect of diet on the CRADs of human gut microbiomes, we analyzed the microbiome data published in a dietary intervention study, in which individuals were fed either an animal-based or a plant-based diet [3]. Analysis of the publicly available 16S sequences revealed that the average *β* values of 0.92 and 0.91 were obtained for the animal and plant dietary intervention, respectively (S1 Table). The ANOVA showed that the *β* values were not significantly different between the dietary intervention samples in the both studies, respectively (P = 0.91 and P = 0.12) (S3 Fig and S2 Table). Thus, similar *β* values of CRADs were observed even between the samples showing changes in microbial abundance due to dietary differences, suggesting that the CRAD of human gut microbiomes was robust and was not largely influenced by dietary changes.

#### CRADs of several human microbiomes of patients with dysbiosis

Many of the gut microbiomes of disease-afflicted patients can have aberrant species composition or richness, a phenomenon called dysbiosis. We compared the CRADs of the gut microbiomes of patients with type 2 diabetes, inflammatory bowel disease (IBD), and multiple sclerosis (MS) with those of corresponding healthy controls [26–29]. The CRADs had similar *β* values of ∼0.91 between these healthy and disease samples with no significant difference by ANOVA (P>0.01) (S4 Fig and S2 Table). These data also suggested that the dysbiotic and normal gut microbiomes shared a common mechanism of CRAD formation, and that the CRAD was not largely affected by changes in host physiological states. However, we cannot exclude the possibility that this is not the case for gut microbiomes of other diseases.

### Variations in CRAD

#### CRADs of microbial communities in the intestinal contents of mice

**Fig 3:**
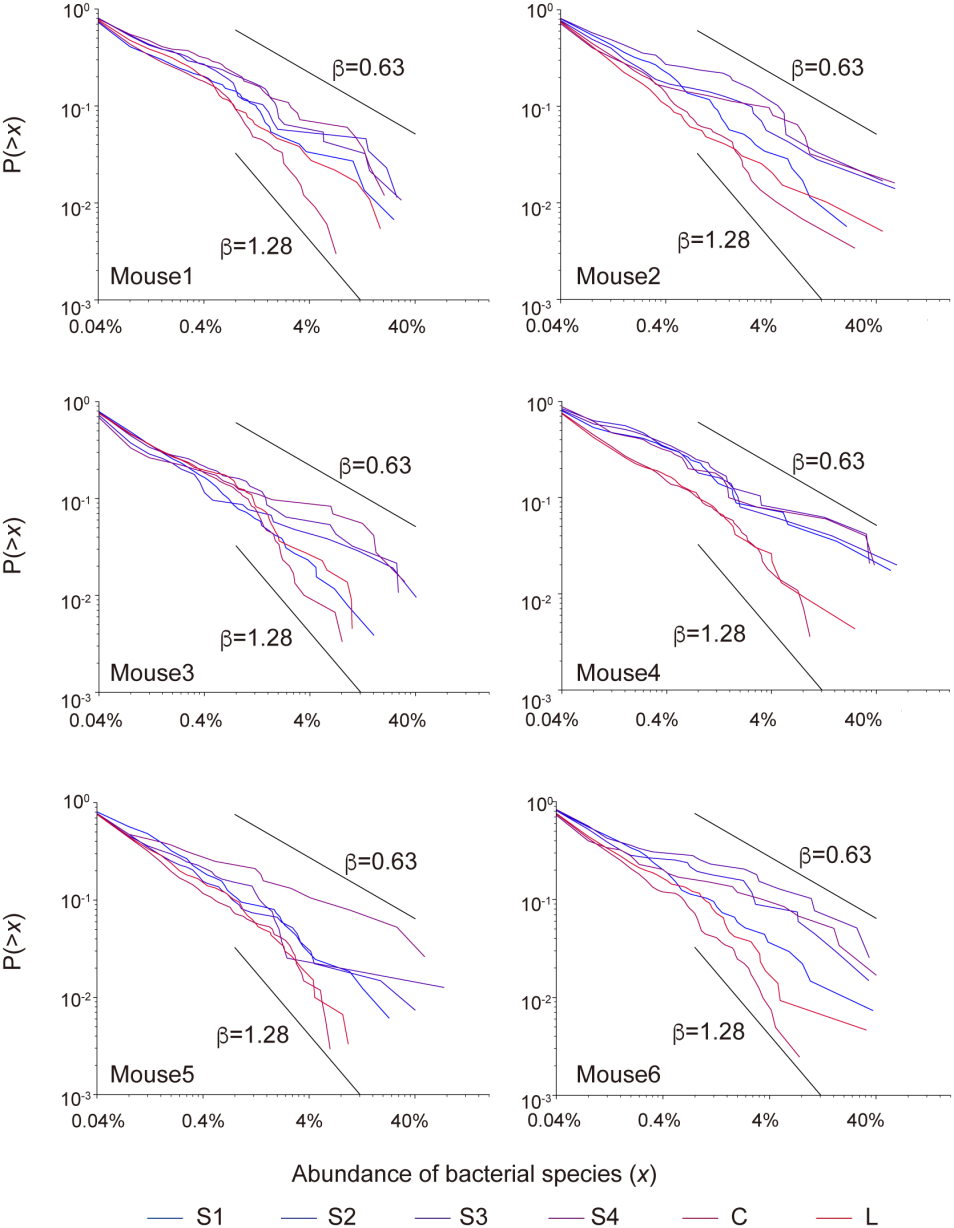
CRADs of the dissected mouse gut contents. S1-S4, C, and L indicate the contents of the small intestine, the cecum, and the large intestine, respectively.

As shown above, the gut microbiomes of mice examined were characterized by similar CRADs with a *β* of 0.89. To identify cases in which *β* shifts to other values, we analyzed the microbial communities in the contents of the large and small intestines of SPF mice. We obtained 16S V1-V2 sequences from the contents of four samples from each of the small intestine, the cecum, and the large intestine (S5A Fig). The 16S analysis revealed that the species richness was varied among the three intestinal regions, and was significantly lower in all four samples from the small intestine than those from the cecum and large intestine (S1B Fig). The average *β* values for the CRADs of the four small intestine samples ranged from 0.62 to 0.88 with the overall average of 0.75, whereas the larger *β* values (1.04 and 1.28 in average) were obtained for the large intestine and cecum samples, respectively (Fig 3 and S1 Table). The ANOVA showed that the similarity of *β* values between the samples from the small and large intestine was significantly different (S2 Table). We also found the positive correlation of the *β* values with the species richness and the Shannon index of these murine microbiome samples (S6A Fig). Furthermore, we theoretically confirmed that the estimated *β* values were positively correlated with the OTU number and the Shannon index by assuming the CRADs following power law (S1 Appendix) (S6B Fig). These data suggested that the communities with relatively small *β* value following power law tend to have the relatively low species richness and evenness of the microbial abundance.

#### CRADs of human infant gut microbiomes

**Fig 4:**
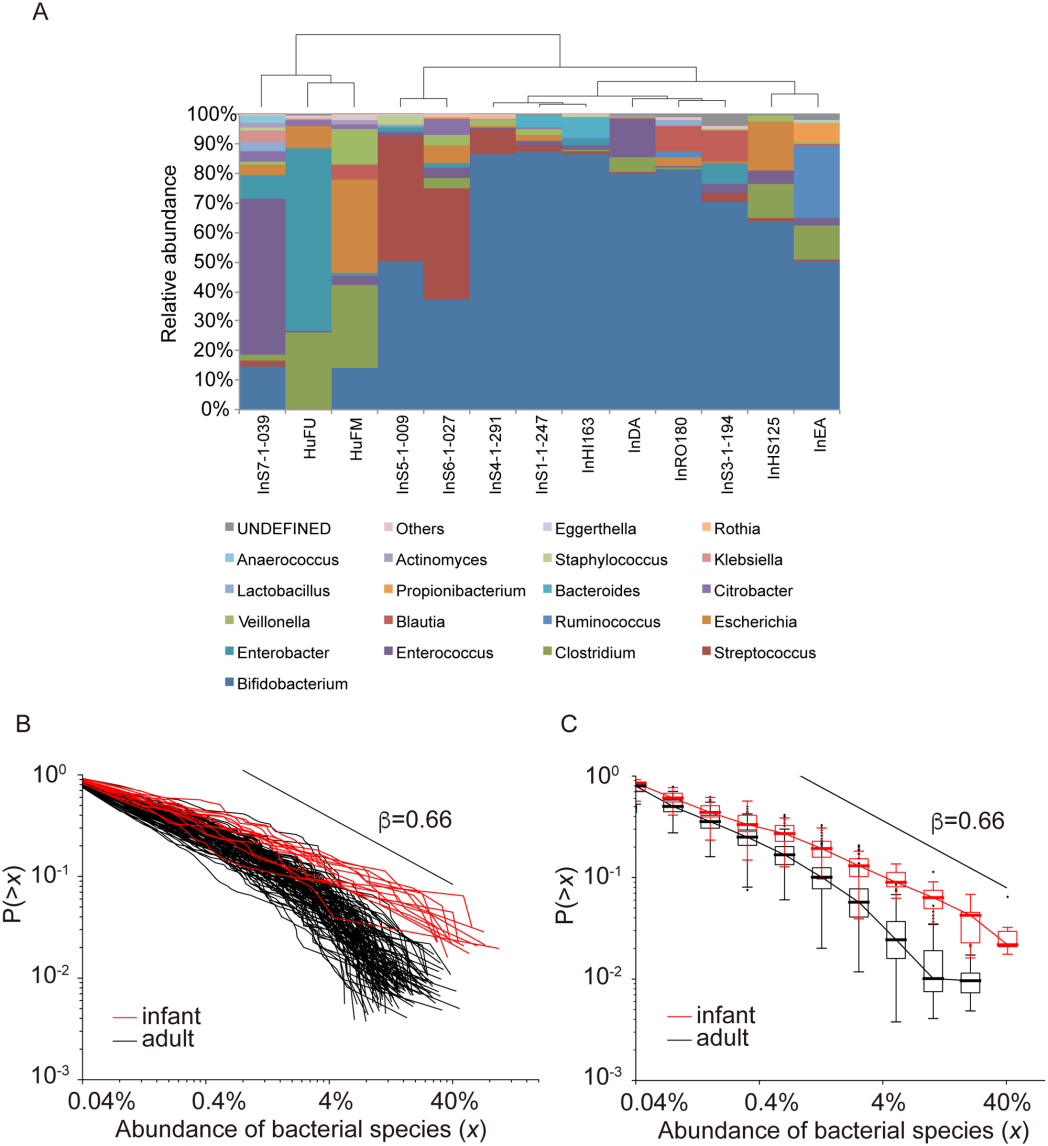
CRADs of the human infant gut microbiome. A: Clustering of the 13 human infant samples at the genus level. Hierarchal clustering is performed at the genus-level abundance of the human infant samples. Each genus is shown in a different color. B: Individual CRADs of gut microbiomes of 13 human infants based on the OTU-level composition. The CRADs of the gut microbiomes of the 13 infants (red) and the 104 healthy Japanese adults (black) are shown. C: Median CRADs of the same samples as those in (B). The CRADs of the gut microbiomes of the 13 infants (red) and the 104 healthy Japanese adults (black) are shown in the boxplot.

We also examined CRADs of the gut microbiomes of human infants of which the species richness is significantly lower than that of the adult gut microbiome [30]. We obtained 16S V1-V2 sequences from fecal samples collected from 13 infants (1 week ∼8 months after birth) in this study. The infant gut microbiomes had considerable interindividual variations with low species richness (Fig 4A, S7 Fig). Most of the *β* values of infant CRADs were significantly different from those of the adult’s CRADs by ANOVA (P<0.01) (S2 Table), and the average *β* value was ∼0.66 (Fig 4B and 4C, and S1 Table), which was similar to that of the CRADs for the mouse small intestine contents. Additionally, we calculated the CRADs for longitudinal gut microbiome samples from newborns to infants of ∼3 years of age by using the published 16S data [31]. These infant samples showed drastic structural changes in species richness and abundance over time (S8A and S8B Fig). The CRADs in the first 100 days after birth had a *β* of 0.66 in average, and over the 3 years, the *β* values gradually reached those of the adults (∼1.03) (S1 Table and S8C Fig). The theoretically estimated *β* values of infant samples were also significantly correlated with the OTU number assuming the CRADs following power law (S1 Appendix, S9 Fig). The smaller *β* values in the CRADs of the microbiomes of human infants and the small intestines of mice suggested that the mechanism for CRAD formation of these communities differed from that for adult microbial communities of human and mouse.

#### CRADs of gut microbiome in antibiotic-treated mice

We also analyzed the fecal microbiome of mice treated with antibiotics, as an additional sample with dramatically reduced species richness [32]. Analysis of the published 16S data showed that the microbial composition of the gut microbiome of antibiotic-treated mice differed from that of untreated controls, and restored to a baseline level similar to that of the controls approximately 2 months after antibiotic treatment ceased (S10A Fig). The gradient of the CRADs of the antibiotic-treated mice samples were even shallower than those of the CRADs of the samples from human infants and the small intestines of mice; they formed an S-shape in the samples during the treatment, and became similar to those of the controls in the samples at approximately 2 months (S10B Fig). These results suggested that the CRADs of antibiotics-treated gut microbiomes were generated by a process distinct from that in human infants and the small intestines of mice. However, increased number of samples would be required to statistically ensure the difference between them because of insufficient number of samples in the dataset.

### Mathematical model and simulation

**Fig 5:**
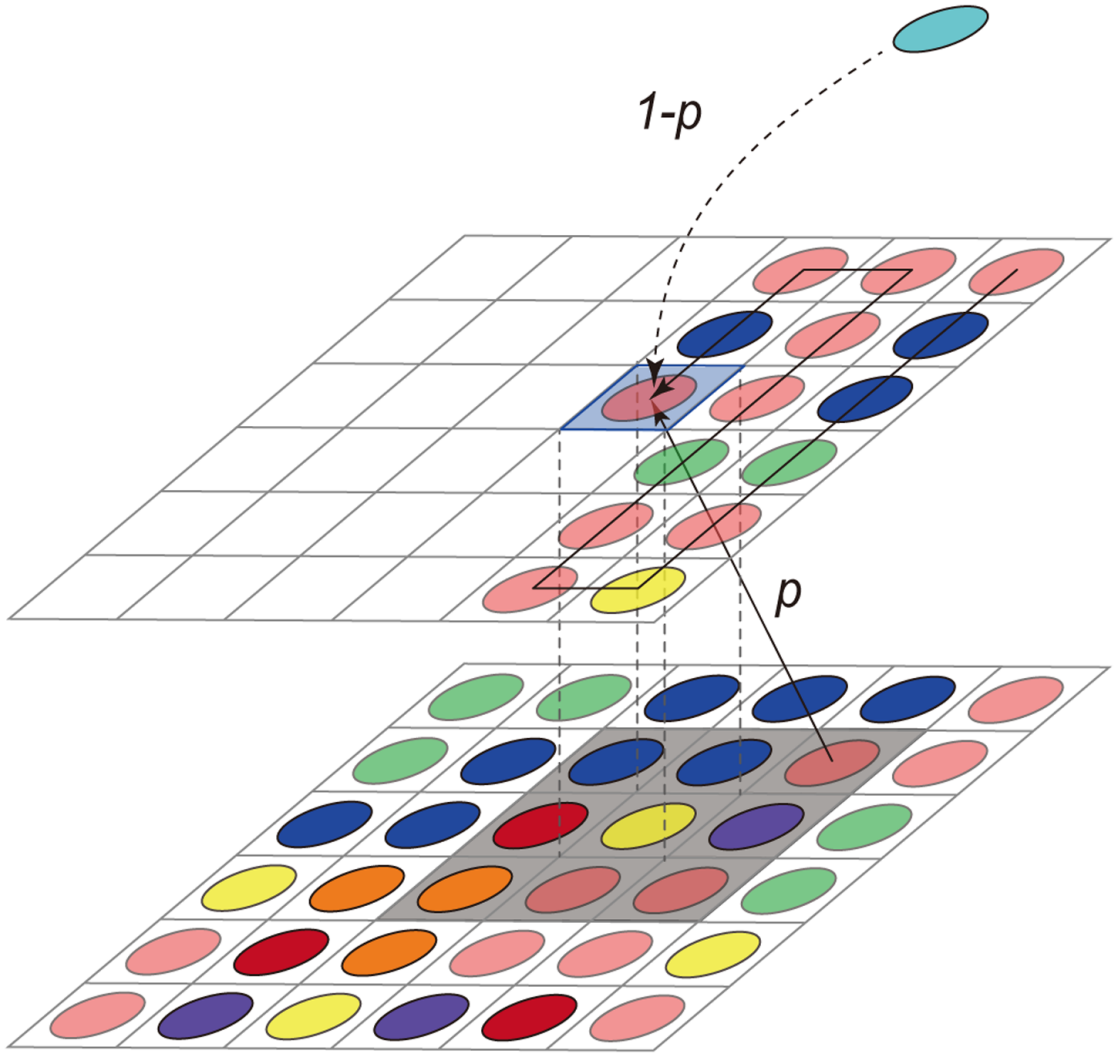
Concept of the mathematical model. To determine the species in the blue-shaded lattice (top), a species in the black shaded area (underneath) is randomly selected with probability *p*. A new additional species joins with probability 1-*p*.

To interpret the variations observed in the CRADs, we developed a 3-dimensional mathematical model for the microbial proliferation process. To design the mathematical model, we first considered that amplification of subtle stochastic variances in proliferation among microbes largely contributed to shaping the gut microbiome because the gut microbiome structure is highly variable, and shows individual variation even between monozygotic twins [33]. We then considered the microbial spatial distribution in the digestive tract. To obtain this information, we performed fluorescence in situ hybridization (FISH) of the mouse small and large intestines to determine the spatial distribution of the microbes, many of which congregated near the mucus layer (S11 Fig), as reported previously [34, 35]. Using these data in addition to the robust CRAD observed in the various gut microbiomes, our mathematical model mainly assumed amplification of stochastic fluctuations in spatially competitive proliferation of microbes congregated near nutrients, without considering microbial growth rate or interactions. We regarded the *z* = 0 plane as the surface of a lump of nutrients to which microbes were attached, and we distributed N(0) species uniformly at random as an initial boundary condition. One microbe was assumed to occupy one lattice point such as a nutrient patch, and the coordinates (*x*, *y*, *z*) on the *z*-th plane were determined in two ways. With a given probability *p*, a microbe was selected from underneath the plane (*x*, *y*, *z*-1) among nine neighbors on the (*z*-1)-th phase and proliferated to the lattice point at (*x*, *y*, *z*) (Fig 5). For the immigration probability, 1-*p*, we considered that a new additional species also immigrated to the lattice point simultaneous with the existing species. This stochastic proliferation process was repeated until all lattice points were occupied by microbes. The simulation time scale is supposed to be several hours to several days by considering the average doubling time of bacterial cells to be of a few hours, and the simulation size could be a dozen micrometers to a few millimeters in the actual size in the intestine. We also supposed that the host mucus as bacterial feed is continuously produced [36] and the intestinal contents are flowing through the digestive tract [37, 38], thus the bacteria continuously compete for the nutrients and are washed out from the intestine. Simulated CRADs were generated using 2,500 randomly selected species, to obtain data comparable to the CRADs calculated using the 16S data (Supplementary methods).

To optimize the model, we first assessed the lattice size in the case of probability *p* = 1, and found that the lattice size (*x*, *y*, *z*) = (40, 40, 2000) generated CRADs with the highest linearity in the log-log plot (S12 Fig). Second, we found that a change in the initial species number did not strongly affect the *β* values (S13 Fig). Third, we assessed variations in probability *p*, in which the *β* values increased when *p* was smaller than 1, indicating that the immigration probability is considered when *p* is set to <1 (S14 Fig). Fourth, we observed that an uneven initial distribution of microbes on the *z* = 0 plane generated a power law CRAD, while an even initial distribution of microbes generated an exponential CRAD (S15 Fig), indicating that the observed CRADs were generated by proliferation of the initial unevenly distributed microbes, as shown in the FISH analysis.

#### Reproduction of the observed CRADs by simulation

**Fig 6:**
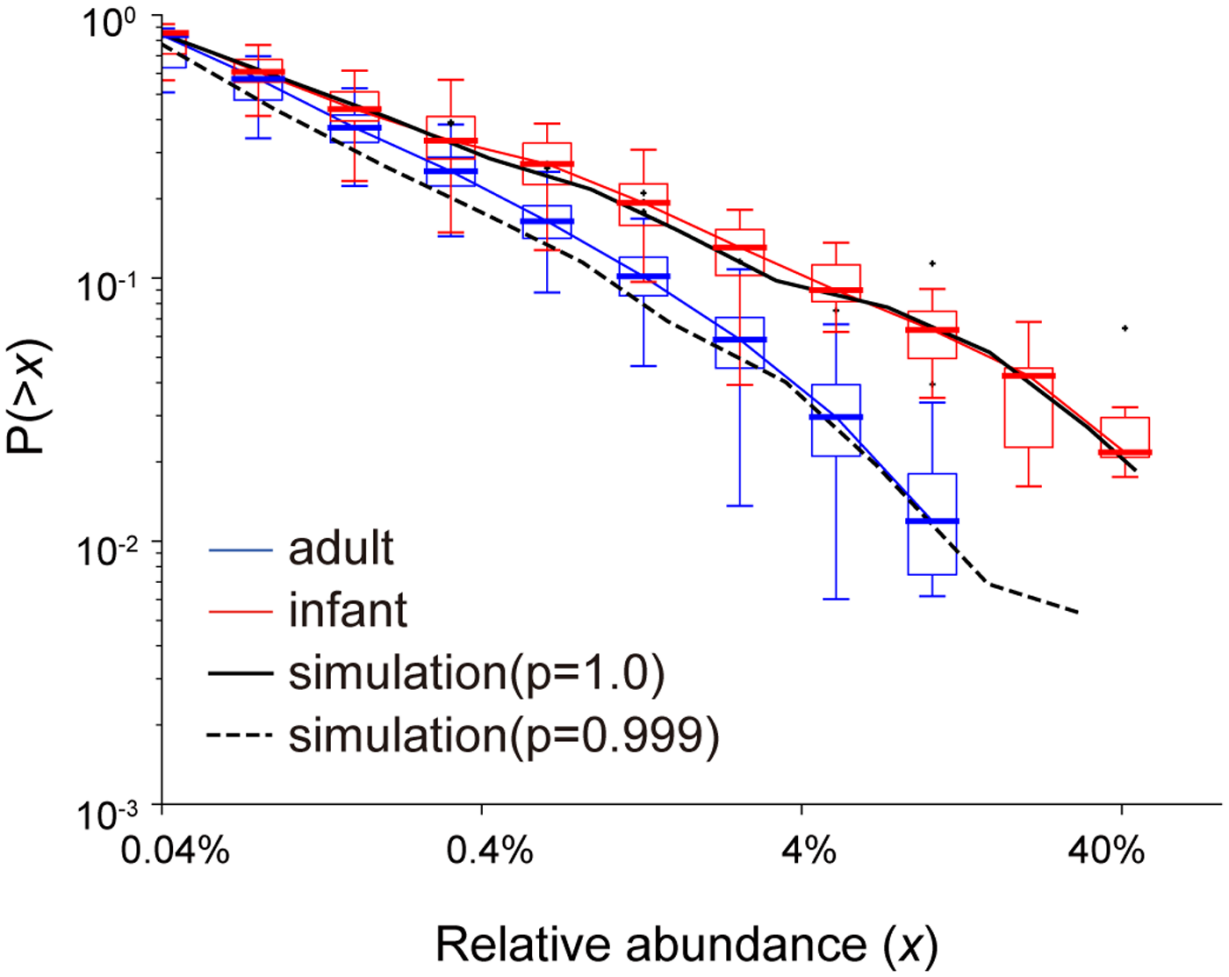
Simulation of CRADs of infant and adult gut microbiomes. Red and blue boxes represent the CRADs of the gut microbiomes of the 13 infants and the 104 Japanese adults, respectively. The solid line and the dashed line represent the simulation results for *p* = 1.0 with 100 initial species and *p* = 0.999 with 200 initial species, respectively. Lattice size in the following simulations was (*x*, *y*, *z*) = (40, 40, 2000). The simulation results are obtained from the average of 100 repetitions.

The observed CRADs of the gut microbiomes of the 104 Japanese adults were reproduced by the simulation under conditions of *p* = 0.999 with an initial species number of 200 in our model without a statistical difference by ANOVA (P>0.01, S3 Table) (Fig 6). In addition, the simulation with *p* = 1.0 and an initial species number of 100 also clearly reproduced the CRAD with a *β* of 0.62, similar to those of the human infant gut microbiomes by ANOVA (P>0.01, S3 Table) (Fig 6). The resulting species numbers in the simulations were 51.0 (*p* = 1) and 189.34 (*p* = 0.999) on average, respectively, which were in the range of the observed species/OTU number in the infant (17∼87) and adult (65∼222) samples. We also obtained the significantly high similarity between the observed and simulated the *β* values in the infant gut microbiomes by ANOVA (P>0.01, S3 Table). The consistency of these simulations strongly supports the appropriateness of our model, and suggested that only existing microbes were involved in the competitive proliferation in the infant gut microbiome, whereas other microbes in addition to the initially present microbes also participated in the competitive proliferation in the adult gut microbiome. The difference in the competitive proliferation process between the infant and adult gut microbiomes may be partly due to differences in diet. Infants’ diets, such as breast milk, contain limited nutrients, which may prevent additional microbes from participating in the competition, while adults’ complex diets, rich in a variety of nutrients, may allow additional microbes to participate in the competition.

**Fig 7:**
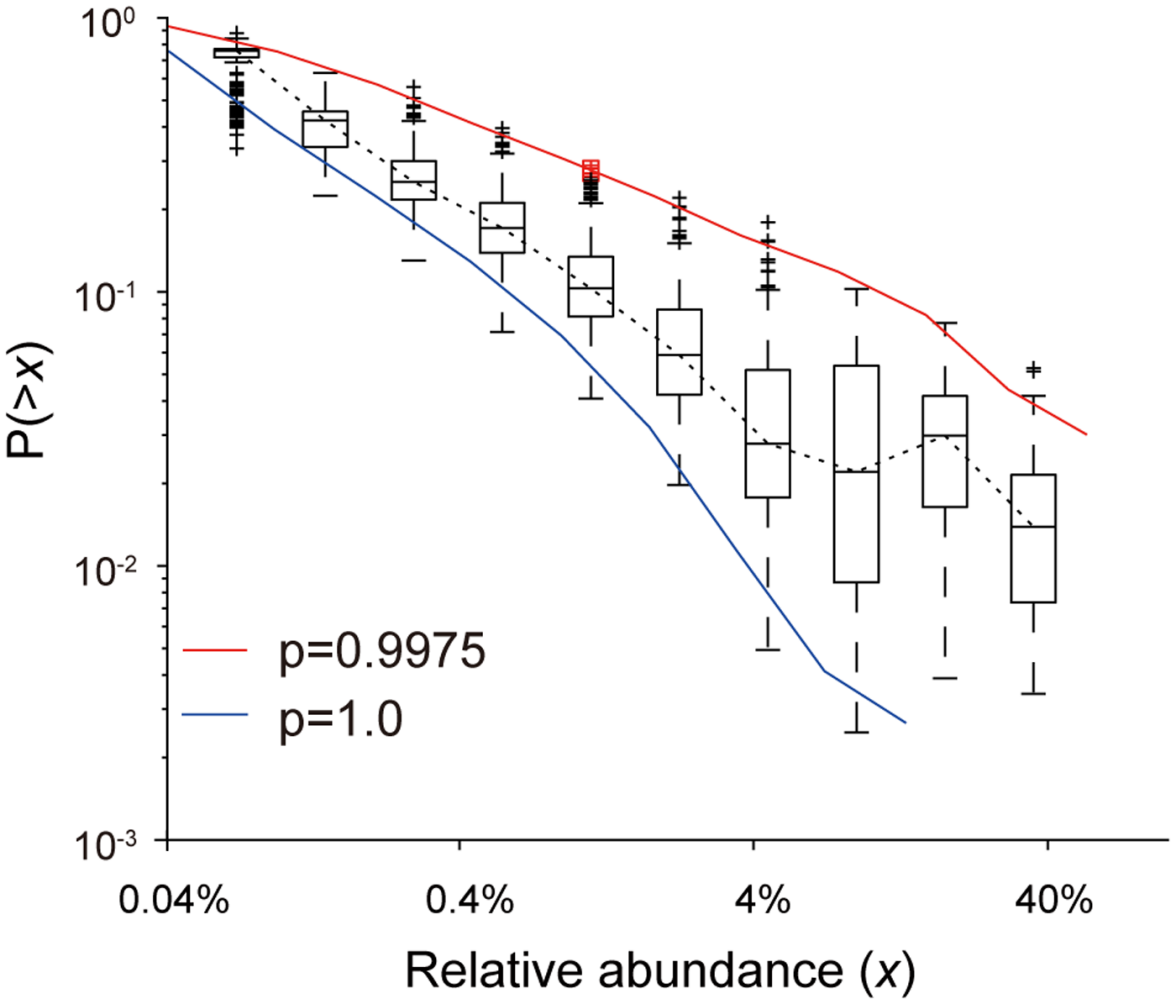
Simulation of CRADs of mice microbiomes. The boxplot shows the observed CRADs of the combined samples, small and large intestine and cecum. Red and blue lines represent the average CRADs in the simulation with *p* = 1.0 and *p* = 0.9975, respectively, with 200 initial species. Lattice size in the following simulations was (*x*, *y*, *z*) = (40, 40, 2000). The simulation results are obtained from the average of 100 repetitions.

For the mouse samples, the simulation using *p* = 1 with 40 initial species also reproduced the CRADs with a small *β* observed in the mouse small intestine by ANOVA (P>0.01, S3 Table) (Fig 7). Furthermore, the CRADs generated by the simulation using *p* = 0.9975 with 200 initial species well matched with the observed CRADs of the cecum contents by ANOVA (P>0.01, S3 Table). The resulting species numbers in the simulations were 33.2 (*p* = 1) and 378.1 (*p* = 0.9975) in average, respectively, which were similar to the smallest and largest species numbers observed in the intestinal content samples of mice (38 and 376, respectively). These results suggested that only existing bacteria participated in the competitive proliferation in the mouse small intestine, similar to the human infant gut microbiome. However, since the small intestine may be rich in nutrients, unlike the human infant gut, participation of additional microbes could be suppressed by the harsh conditions, such as the high bile acid levels in the small intestine compared with the large intestine.

#### Reproduction of CRADs in the antibiotics-treated gut microbiomes in mice by simulation

**Fig 8:**
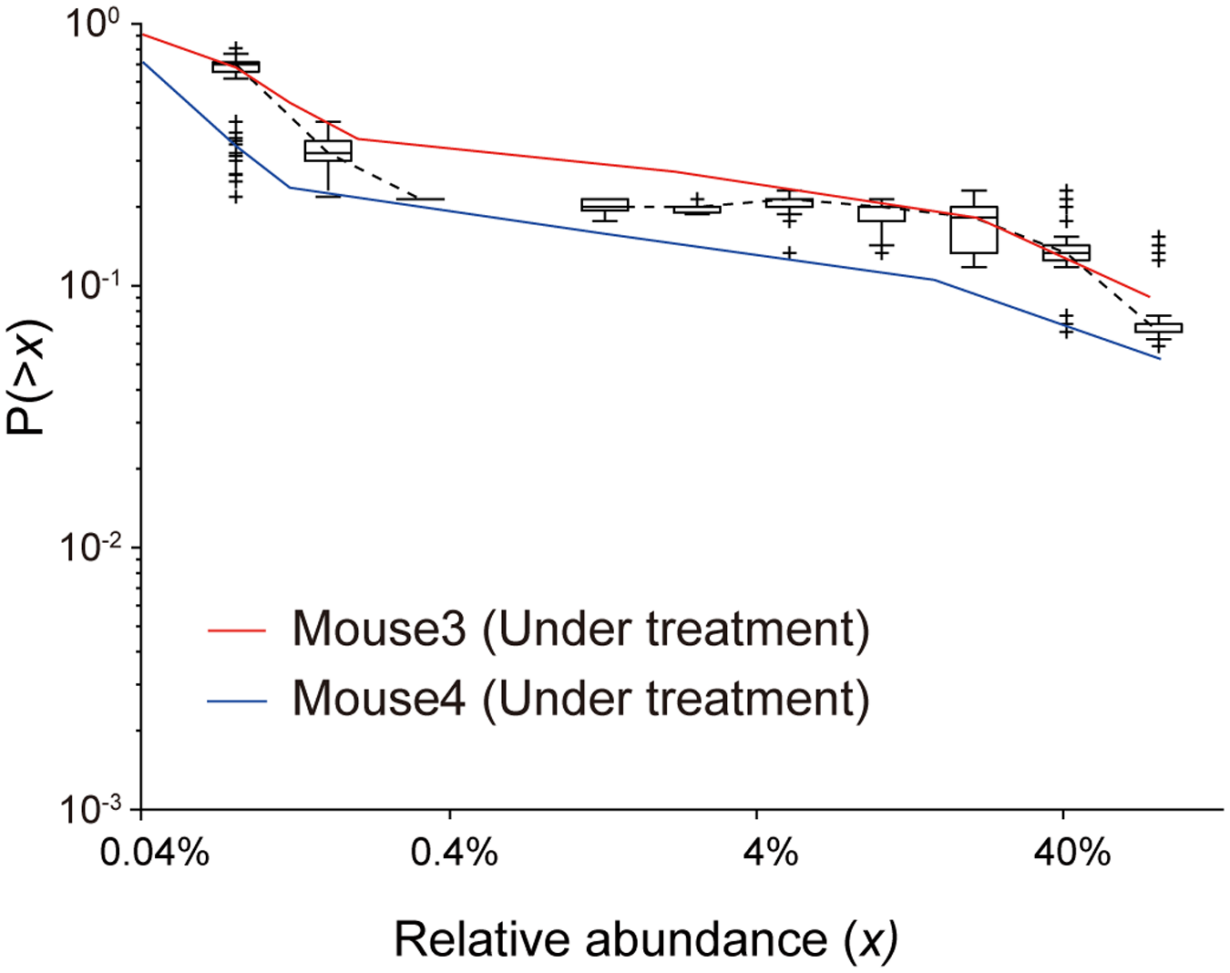
Simulation of CRADs of gut microbiomes in antibiotic-treated mice. The red and blue lines represent the CRADs of samples from two mice treated with antibiotics. Black boxplots represent the CRADs in the simulation with *p* = 1, where the growth rate of 197 of the 200 species was set to half that of the other 3 species. Lattice size in the following simulations was (*x*, *y*, *z*) = (40, 40, 2000). The simulation results are obtained from the average of 100 repetitions.

For the CRADs with a very shallow gradient observed in mice treated with antibiotics, we assumed that the growth rate was not uniform among microbes. For example, when we assumed that the probability of proliferation of 197 of the species was half that of the other 3 species in the initial 200 species in the simulation, the CRADs obtained under this simulation had much shallower gradient than those in the simulation with a uniform growth rate, and were similar to the values observed for the antibiotic-treated mouse gut microbiome (Fig 8). These data imply that species that are sensitive and tolerant to antibiotics have different growth rates under an antibiotic treatment, resulting in the altered proliferation in which only limited or biased species participated, which may differ from the proliferation process for the samples of the human infant feces and mouse small intestines with small *β* values. However, we could not perform statistical assessment for these data because of the insufficient number of samples.

## Conclusion

Overall, we found that the CRAD indicated that gut microbiomes exhibiting high interindividual variability show robust community structure. We approximated the CRADs by power law functions and estimated the power exponent by MLE (S1 Table), further suggesting that the CRADs underlie universal rule shaping the gut microbiome, independent of host-associated factors such as lifestyle, physiology, or genetic background. Since most of the CRAD variations observed in the various microbial communities could be explained by our mathematical model developed in this study without considering microbial growth rate or interactions, it can be suggested that spatially competitive proliferation of unevenly distributed microbes is the primary process generating CRADs following power law. In addition, subtle proliferation differences between microbial species were amplified to produce large differences over time in the present model. This property of our model would generate a random change in the ranking of the resulting abundant species, even if similar species are involved in the initial stages of proliferation, which is concordant with the high interindividual variability observed in the gut microbiome even for monozygotic twins [33]. The positive correlation of the *β* value of CRAD with the species richness and the Shannon index that evaluate ecological features of the bacterial community implies that the *β* value of CRAD can also be used as a novel metric for ecological evaluation of the bacterial community. We found that similar *β* values were observed in many of a variety of samples analyzed in this study though it is known that the species richness and diversity index are varied by various factors. This ambivalence might be partly due to involvement of various components in community structure such as bacterial interaction and bacterial growth in the CRADs and *β* values of actual data.

It should be noted that CRAD provides useful information for elucidating the mechanism for shaping of the gut microbiome as shown here. However, we still have a difficulty to explain the ranking fluctuations of microbial species, some of which are commonly or uniquely abundant in the gut microbiome. The heterogeneous bacterial growth rate and their complex interactions might be involved in the ranking diversity of microbes. These parameters will be integrated for developing the finer model in near future when their experimental data are available. Thus, further development of mathematical models will be of use for their application to clinical medicine and food science such as manipulations of the human gut microbiome linking to human health.

## Supporting information

S1 Fig Fitting of CRADs by several heavy-tailed distributions. The data were fitted by maximum likelihood estimation to power law, log-normal, and stretched exponential distributions, respectively.
(TIF)

S2 Fig CRADs from the metagenomic data of the 104 Japanese individuals. The relative abundance of bacterial species is obtained from the paper by Nishijima et al. [24].
(TIF)

S3 Fig CRADs of human gut microbiomes in dietary intervention. The data are from the paper by Lawrence et al. [3]. We used 2,500 high-quality reads of the 16S V4 region from fecal samples from 20 individuals, in which 10 subjects ate an animal-based diet for 4 days, while the other 10 ate a plant-based diet for 4 days. Two samples with very high or very low abundance OTUs were excluded from the data set. A: CRADs of gut microbiomes from individuals with an animal-based diet. Boxplots represent the CRADs before, during, and after the change to an animal-based diet. The horizontal axis indicates the abundance of each species or OTU, and the vertical axis indicates the probability that OTU abundance is greater than the value of the horizontal axis. B: CRADs of gut microbiomes from individuals with a plant-based diet. Boxplots represent the CRADs before, during, and after the change to a plant-based diet. The horizontal axis indicates the abundance of each species or OTU, and the vertical axis indicates the probability that OTU abundance is greater than the value of the horizontal axis.
(TIF)

S4 Fig CRADs of normal and altered gut microbiomes with dysbiosis. A: CRADs of patients with multiple sclerosis (MS). The boxplots indicate the CRADs of healthy individuals and patients with MS. Data are from the paper by Miyake et al. [29]. B: CRADs of patients with inflammatory bowel disease (IBD). The boxplots indicate the CRADs of healthy individuals and patients with IBD. Data are from the paper by Qin et al. [28]. C: CRADs of Swedish patients with type 2 diabetes (T2D). The boxplots indicate the CRADs of healthy individuals and patients with type 2 diabetes. Data are from the paper by Qin et al. [26]. D: CRADs of Chinese patients with type 2 diabetes (T2D). The boxplots indicate the CRADs of healthy individuals and patients with type 2 diabetes. Data are from the paper by Karlsson et al. [27].
(TIF)

S5 Fig Dissected sections of mice gut. For each sample, 2,500 high-quality 16S V1-2 sequences were analyzed. A: Dissected sections of mouse gut. The intestines of six SPF mice were dissected into six sections and the intestinal contents were collected. B: OTU number of each dissected section of mouse gut. OTU numbers generated by clustering of the 16S reads from each dissected section from each mouse are shown. Each color indicates a different mouse.
(TIF)

S6 Fig Correlation of *β* with species richness and diversity in dissected mice intestinal samples. A: Correlation of *β* with species richness and diversity in dissected mice intestinal samples of mice. Spearman’s rank correlation coefficient was calculated between *β* values and the observed OTU numbers and the Shannon indices. B: Theoretical analysis of correlation of *β* with species richness and diversity. Theoretically predicted OTU numbers and Shannon index using the estimated *β* values of the observed samples were plotted.
(TIF)

S7 Fig OTU number of gut microbiomes collected from 13 human infants. The left boxplot shows the OTU numbers of feces collected from 13 infants, and the right boxplot shows the OTU numbers of feces collected from 104 healthy Japanese adults.
(TIF)

S8 Fig CRADs of longitudinal gut microbiomes of human infants. Data are from the paper by Koenig et al. [31]. For each sample, 2,500 high-quality 16S V4 sequences were analyzed. A: Relationship between OTU number and days after birth. The horizontal axis indicates days after birth, and the vertical axis indicates OTU number. B: Bacterial composition at the genus level. The bacterial composition at the genus level based on 16S data is shown in the bar graph. C: CRADs of longitudinal gut microbiomes of human infants. CRADs of gut microbiomes from infants at 4, 31, 63, 141, 172, 252, 469, 831 days after birth and that of the mother are shown.
(TIF)

S9 Fig Correlation of *β* with species richness and diversity in infant samples. A: Correlation of *β* with species richness and diversity in infant samples. We used infant time-course samples for this analysis [31]. Spearman’s rank correlation coefficient was calculated between *β* values and the observed OTU numbers and the Shannon’s indices. B: Theoretical analysis of correlation of *β* with species richness and diversity. Theoretically predicted OTU numbers and Shannon index using the estimated *β* values of the observed samples were plotted.
(TIF)

S10 Fig CRADs of gut microbiomes of antibiotic-treated mice. Data are from the paper by Dollive et al. [32]. Control fecal samples of two mice (mouse 1, 2), another two fecal samples of mice treated with antibiotics for 2 days (mouse 3, 4), and two fecal samples of mice in which antibiotic treatment was stopped (mouse 5, 6) were analyzed. For each sample, 2,500 high-quality 16S V1-V2 sequence data were analyzed. A: Bacterial composition at the genus level. The bacterial composition at the genus level calculated using the 16S data is shown in the bar graph. B: CRADs of antibiotic-treated mice. CRADs of the gut microbiomes of all six mice from the analysis of 16S data.
(TIF)

S11 Fig FISH images of microorganisms in mouse digestive tracts. A: Visualization of the microbial distribution in the small intestine (distal). The sample was stained with Muc2 (green), EUB338 (red), and DAPI (blue). B: Visualization of the microbial distribution in the large intestine. The sample was stained with Muc2 (green), EUB338 (red), and DAPI (blue).
(TIF)

S12 Fig Effect of lattice size on CRADs in the simulation. CRADs for each simulation height (40, 200, 1000, 2000, 4000, 40000) are shown. Initial bacterial cells were filled on the bottom of a 40 × 40 lattice. Initial species number was 200. Immigration probability *p* = 1.
(TIF)

S13 Fig Effect of initial species number on CRADs in the simulation. The colored solid line represents the CRADs of simulations started with different numbers of species (20, 40, 100, 200, 300, 1000). Immigration probability, p, was 1.0. Simulation size was 40 × 40 × 2000.
(TIF)

S14 Fig Effect of immigration probability on CRADs in the simulation. The solid line represents the CRAD of each probability *p* (0.99, 0.995, 0.999, 0.9995, 1.0). Simulation size was 40 × 40 × 2000. Initial species number is 200.
(TIF)

S15 Fig Effect of initial spatial distribution of bacteria on CRADs in the simulation. A: Image of bacterial proliferation in the simulation over time (time t = 0, 500, ∞), with non-uniform initial spatial distribution. Each color represents a different bacterial species. Simulation size was 10 × 10 × 50. Initial species number is 20. B: Image of bacterial proliferation in the simulation over time (time t = 0, 500, ∞), with a uniform initial spatial distribution. Each color represents a different bacterial species. Simulation size was 10 × 10 × 50. Initial species number is 20. C: The solid line represents the CRADs of simulations start with uniform and non-uniform spatial distributions. Simulation conditions are the same as above.
(TIF)

S1 Appendix Species richness and diversity under the power law CRADs. (PDF)

S1 Table Estimation of the average *β* values calculated by MLE in this study. Average *β* values are estimated using the relative abundances between 0.8 and 40% in the CRADs. MLE: Maximum likelihood estimation.
(XLSX)

S2 Table ANOVA assessment of variance between the observed *β* values of given samples. Average *β* values of the relative abundances between 0.8 and 40% in the CRADs are used for this analysis.
(XLSX)

S3 Table ANOVA assessment of variance between the observed and simulated *β* values. Average *β* values of the relative abundances between 0.8 and 40% in the CRADs are used for this analysis.
(XLSX)
