# Supplementary figures and images for: A 3-dimensional mathematical model of microbial proliferation that generates the characteristic cumulative relative abundance distributions in gut microbiomes
Source: PLoS One. 2017 Aug 8;12(8):e0180863. doi: 10.1371/journal.pone.0180863 (PMC5549704; doi:10.1371/journal.pone.0180863)

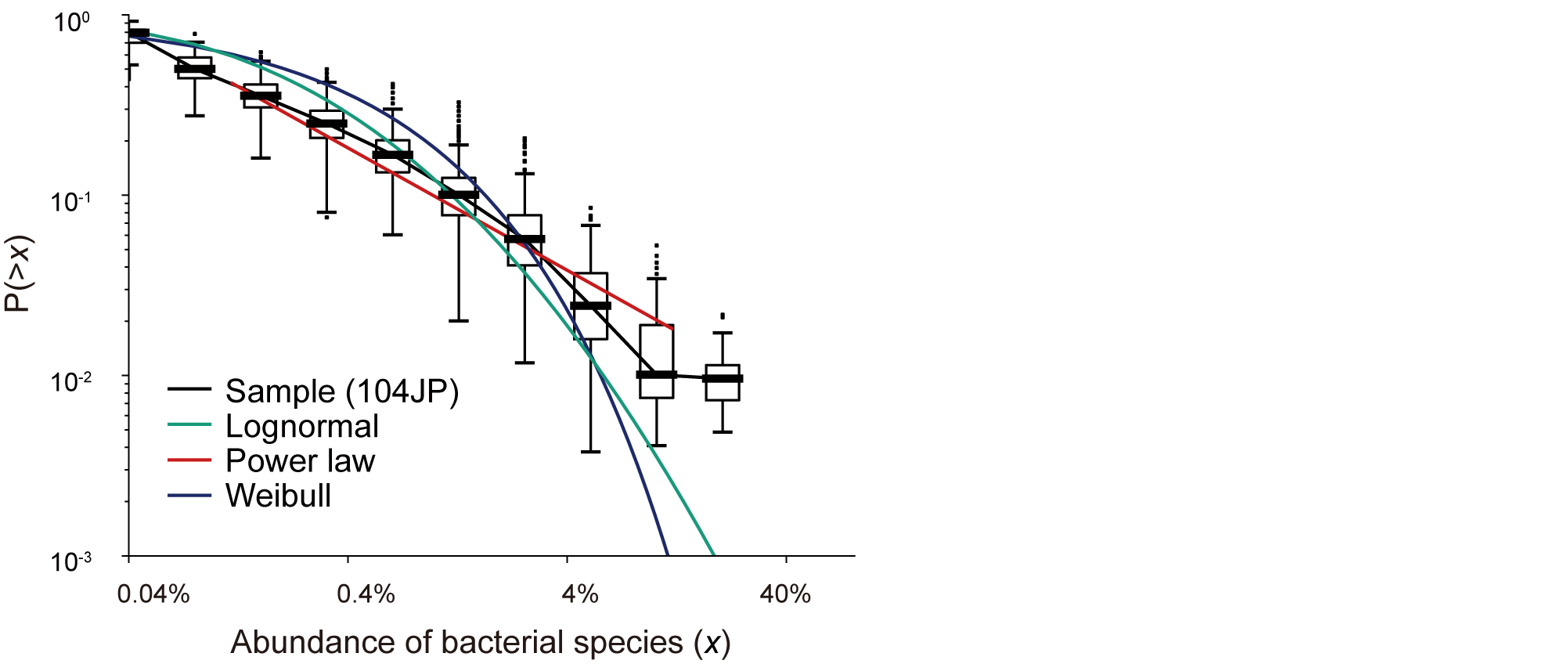

Supplement: S1 Fig — The data were fitted by maximum likelihood estimation to power law, log-normal, and stretched exponential distributions, respectively. (TIF) [file pone.0180863.s001.tif]

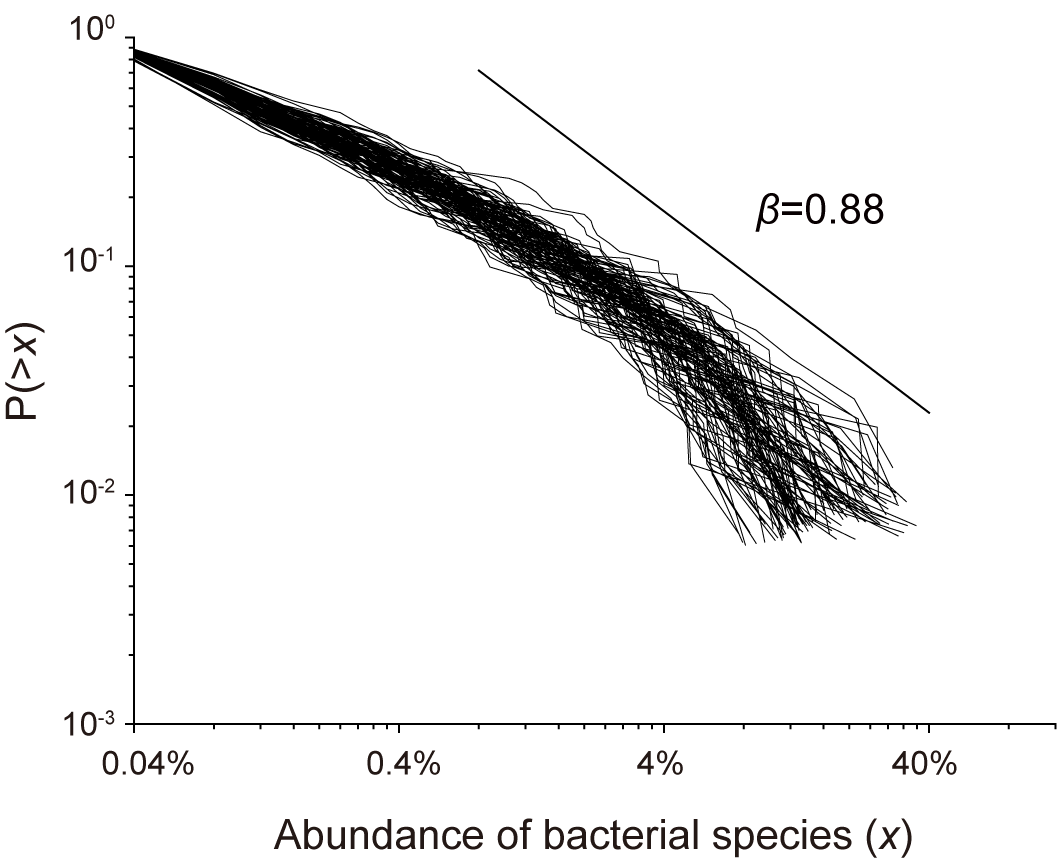

Supplement: S2 Fig — The relative abundance of bacterial species is obtained from the paper by Nishijima et al. [24]. (TIF) [file pone.0180863.s002.tif]

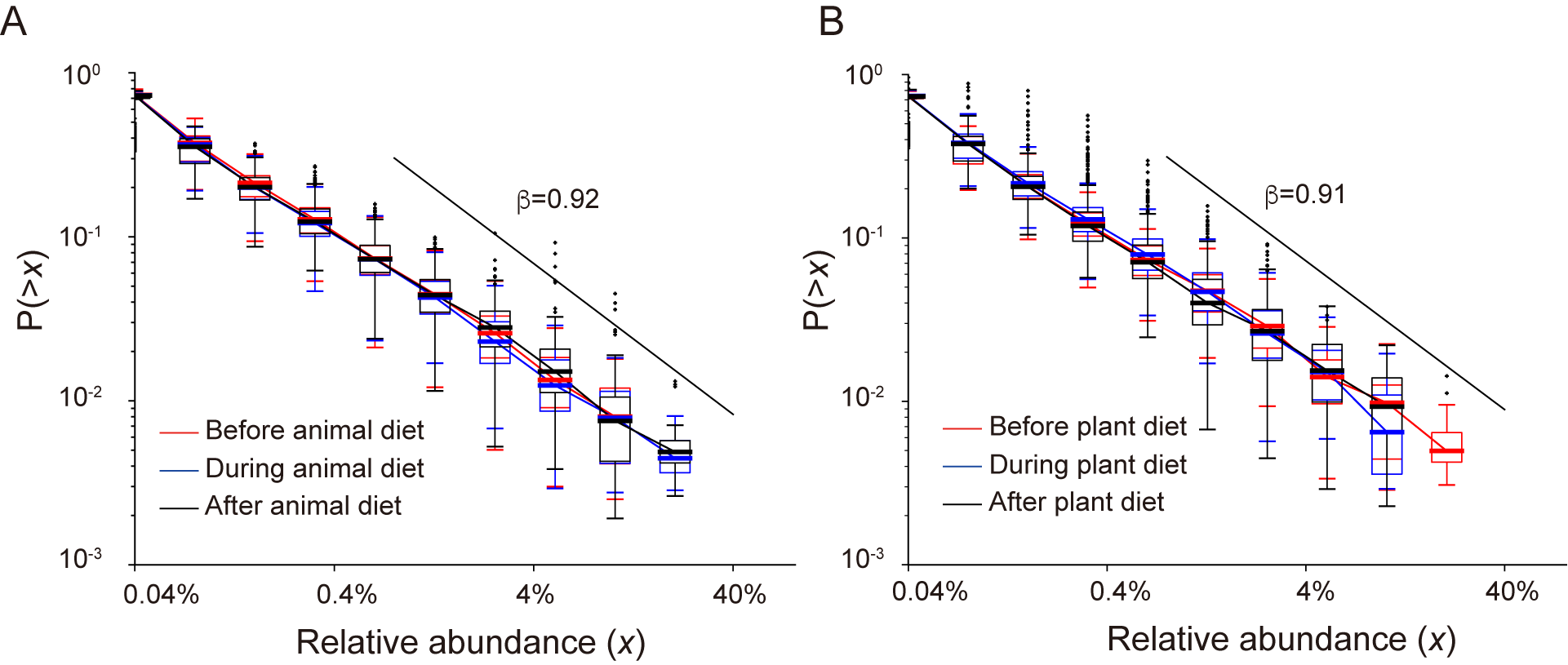

Supplement: S3 Fig — The data are from the paper by Lawrence et al. [3]. We used 2,500 high-quality reads of the 16S V4 region from fecal samples from 20 individuals, in which 10 subjects ate an animal-based diet for 4 days, while the other 10 ate a plant-based diet for 4 days. Two samples with very high or very low abundance OTUs were excluded from the data set. A: CRADs of gut microbiomes from individuals with an animal-based diet. Boxplots represent the CRADs before, during, and after the change to an animal-based diet. The horizontal axis indicates the abundance of each species or OTU, and the vertical axis indicates the probability that OTU abundance is greater than the value of the horizontal axis. B: CRADs of gut microbiomes from individuals with a plant-based diet. Boxplots represent the CRADs before, during, and after the change to a plant-based diet. The horizontal axis indicates the abundance of each species or OTU, and the vertical axis indicates the probability that OTU abundance is greater than the value of the horizontal axis. (TIF) [file pone.0180863.s003.tif]

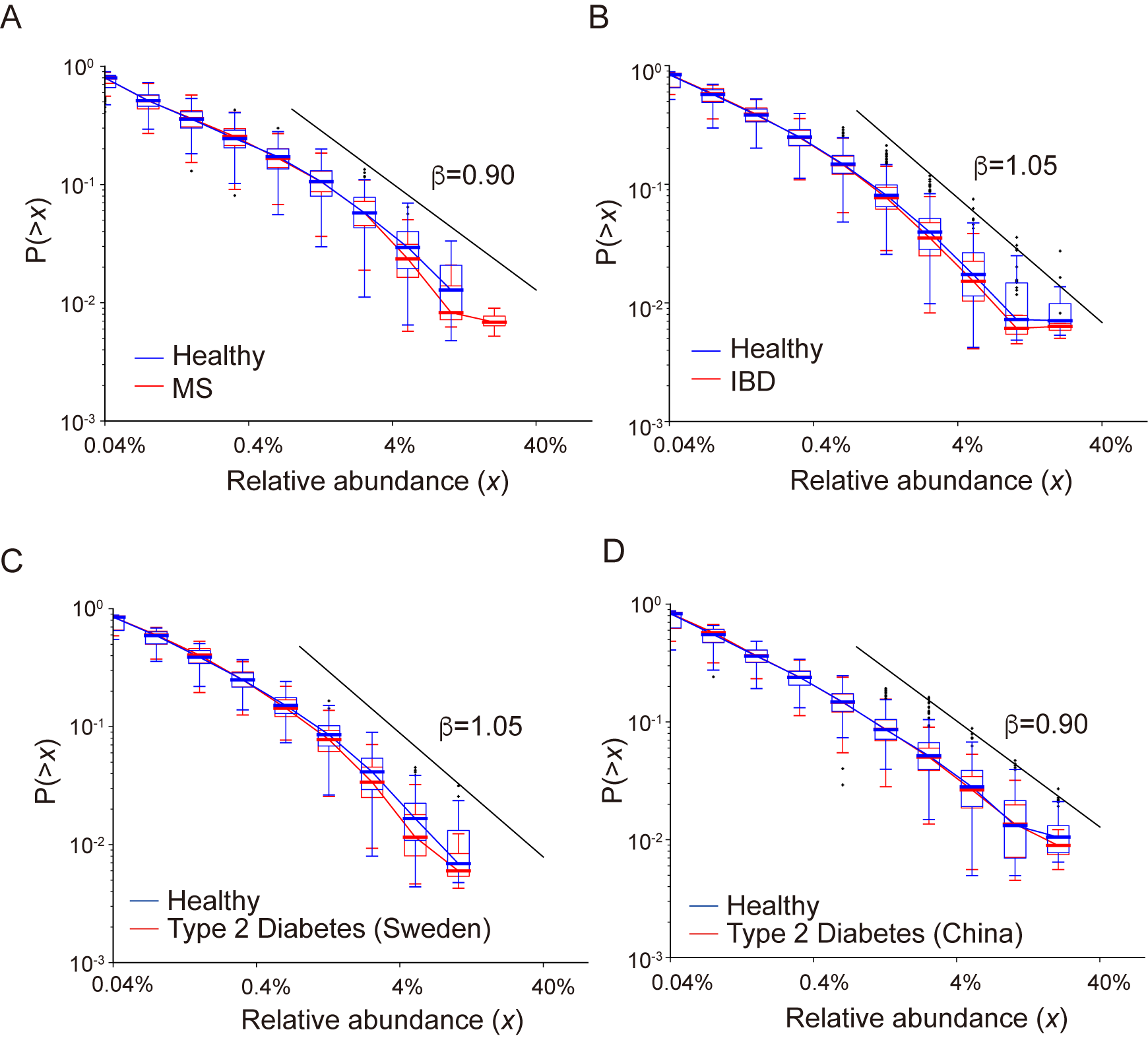

Supplement: S4 Fig — A: CRADs of patients with multiple sclerosis (MS). The boxplots indicate the CRADs of healthy individuals and patients with MS. Data are from the paper by Miyake et al. [29]. B: CRADs of patients with inflammatory bowel disease (IBD). The boxplots indicate the CRADs of healthy individuals and patients with IBD. Data are from the paper by Qin et al. [28]. C: CRADs of Swedish patients with type 2 diabetes (T2D). The boxplots indicate the CRADs of healthy individuals and patients with type 2 diabetes. Data are from the paper by Qin et al. [26]. D: CRADs of Chinese patients with type 2 diabetes (T2D). The boxplots indicate the CRADs of healthy individuals and patients with type 2 diabetes. Data are from the paper by Karlsson et al. [27]. (TIF) [file pone.0180863.s004.tif]

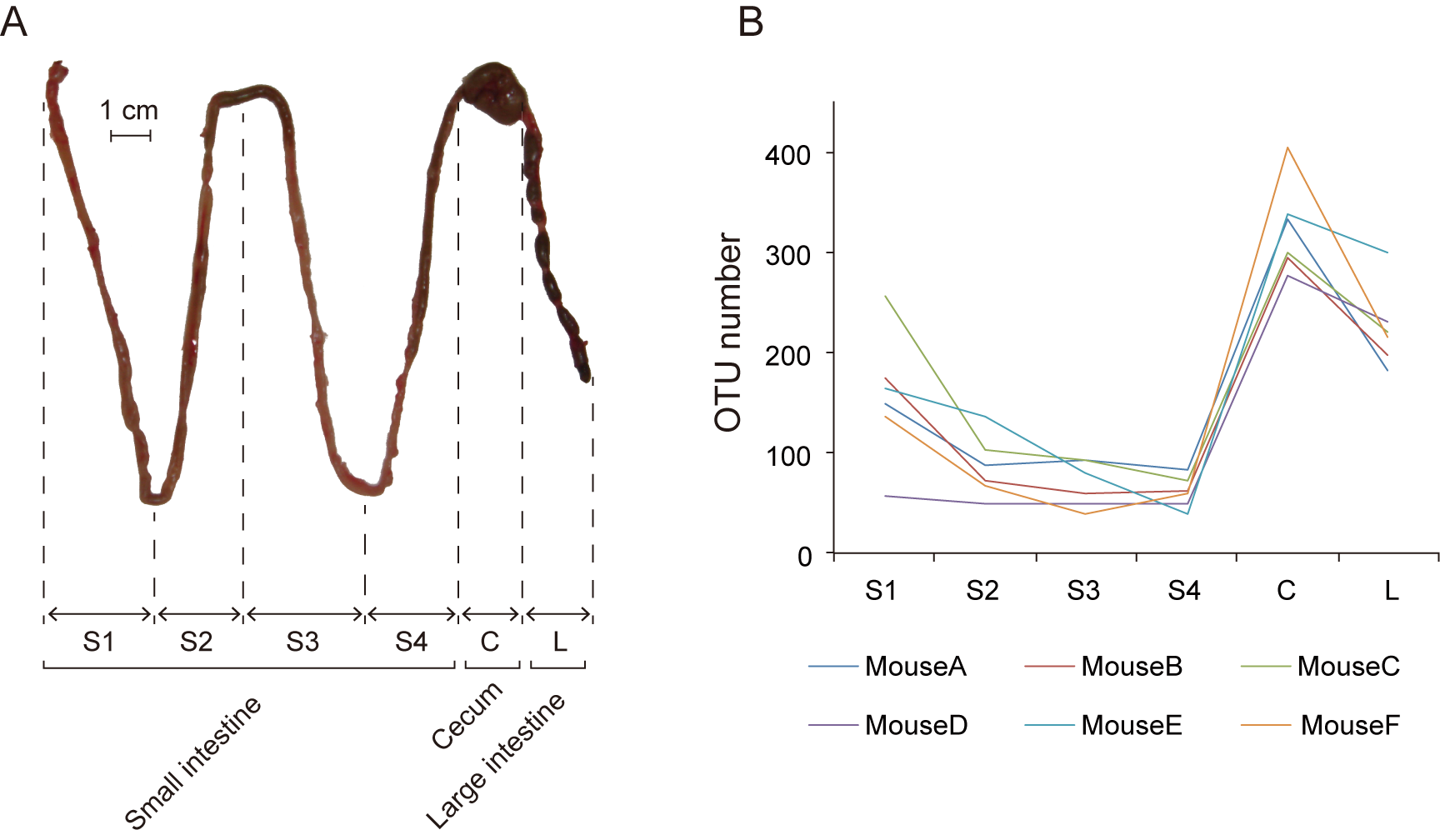

Supplement: S5 Fig — For each sample, 2,500 high-quality 16S V1-2 sequences were analyzed. A: Dissected sections of mouse gut. The intestines of six SPF mice were dissected into six sections and the intestinal contents were collected. B: OTU number of each dissected section of mouse gut. OTU numbers generated by clustering of the 16S reads from each dissected section from each mouse are shown. Each color indicates a different mouse. (TIF) [file pone.0180863.s005.tif]

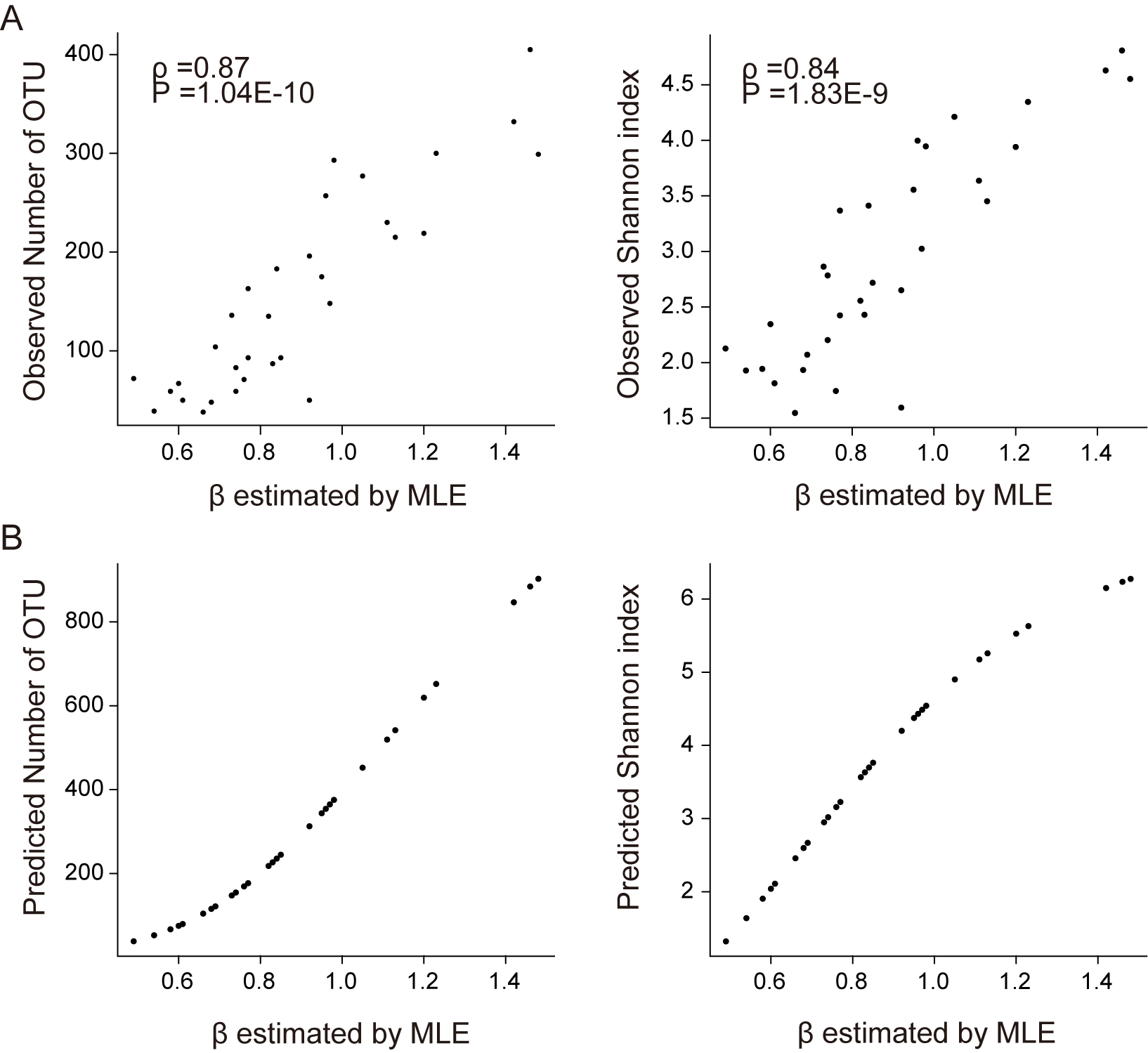

Supplement: S6 Fig — A: Correlation of β with species richness and diversity in dissected mice intestinal samples of mice. Spearman’s rank correlation coefficient was calculated between β values and the observed OTU numbers and the Shannon indices. B: Theoretical analysis of correlation of β with species richness and diversity. Theoretically predicted OTU numbers and Shannon index using the estimated β values of the observed samples were plotted. (TIF) [file pone.0180863.s006.tif]

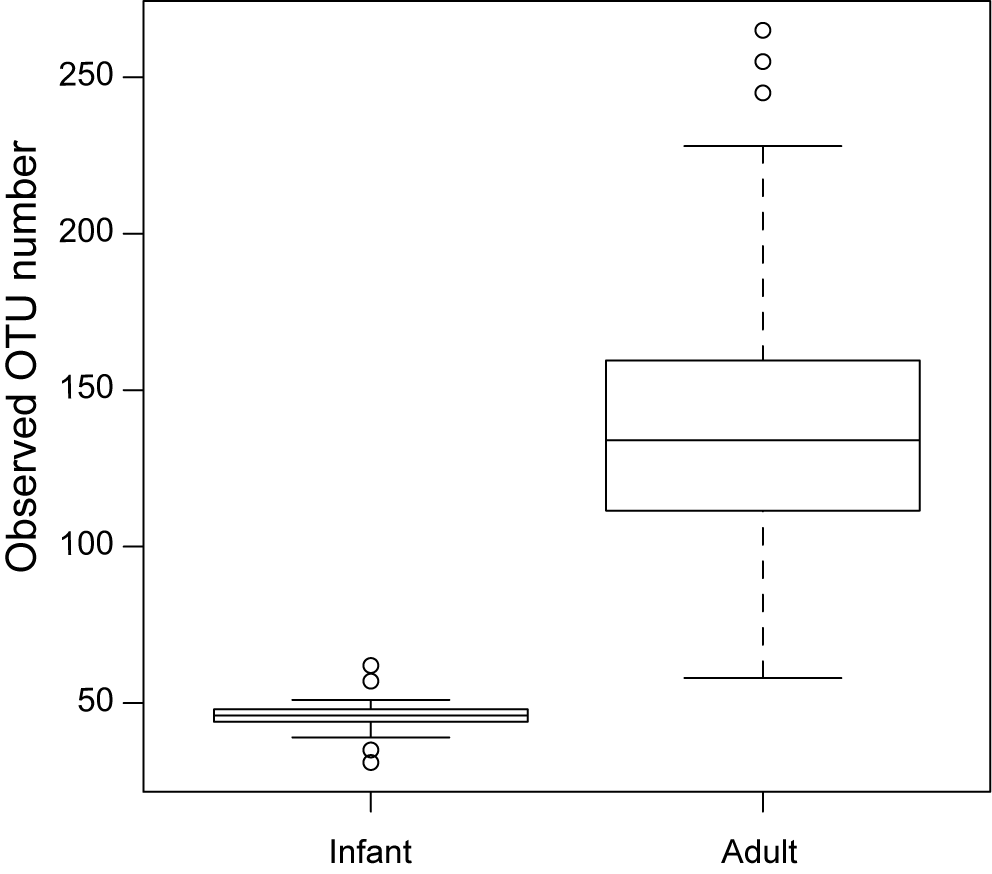

Supplement: S7 Fig — The left boxplot shows the OTU numbers of feces collected from 13 infants, and the right boxplot shows the OTU numbers of feces collected from 104 healthy Japanese adults. (TIF) [file pone.0180863.s007.tif]

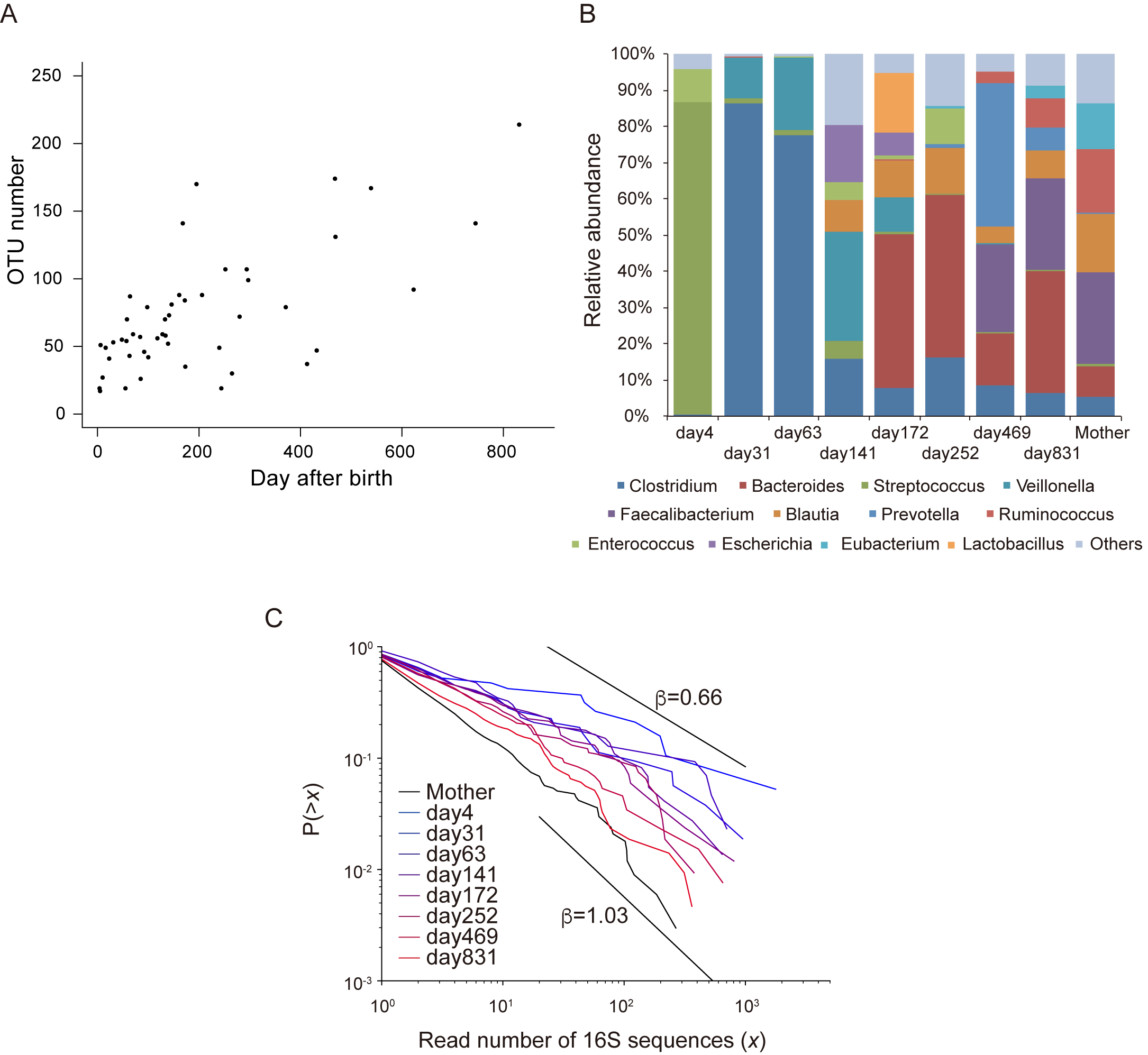

Supplement: S8 Fig — Data are from the paper by Koenig et al. [31]. For each sample, 2,500 high-quality 16S V4 sequences were analyzed. A: Relationship between OTU number and days after birth. The horizontal axis indicates days after birth, and the vertical axis indicates OTU number. B: Bacterial composition at the genus level. The bacterial composition at the genus level based on 16S data is shown in the bar graph. C: CRADs of longitudinal gut microbiomes of human infants. CRADs of gut microbiomes from infants at 4, 31, 63, 141, 172, 252, 469, 831 days after birth and that of the mother are shown. (TIF) [file pone.0180863.s008.tif]

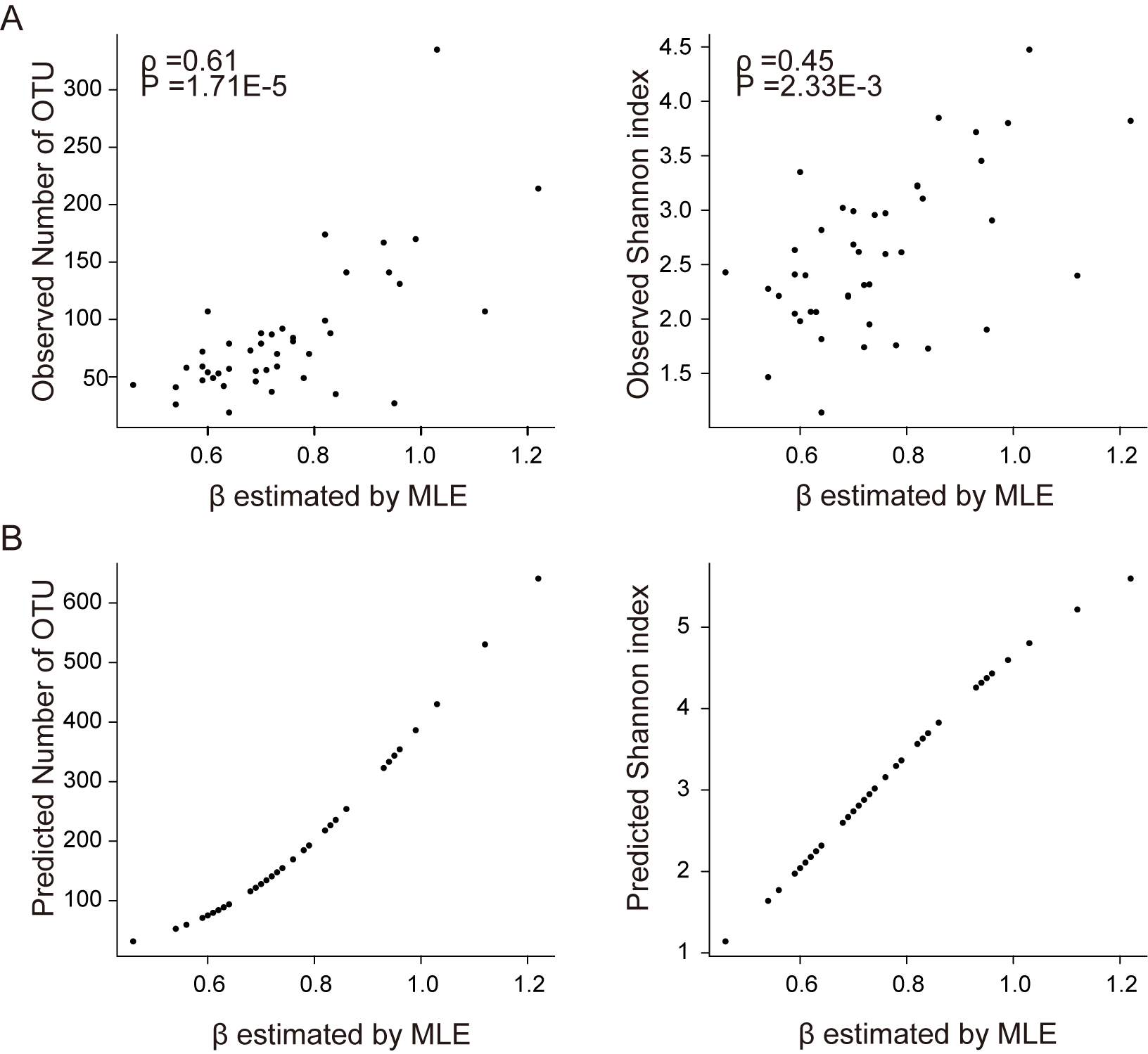

Supplement: S9 Fig — A: Correlation of β with species richness and diversity in infant samples. We used infant time-course samples for this analysis [31]. Spearman’s rank correlation coefficient was calculated between β values and the observed OTU numbers and the Shannon’s indices. B: Theoretical analysis of correlation of β with species richness and diversity. Theoretically predicted OTU numbers and Shannon index using the estimated β values of the observed samples were plotted. (TIF) [file pone.0180863.s009.tif]

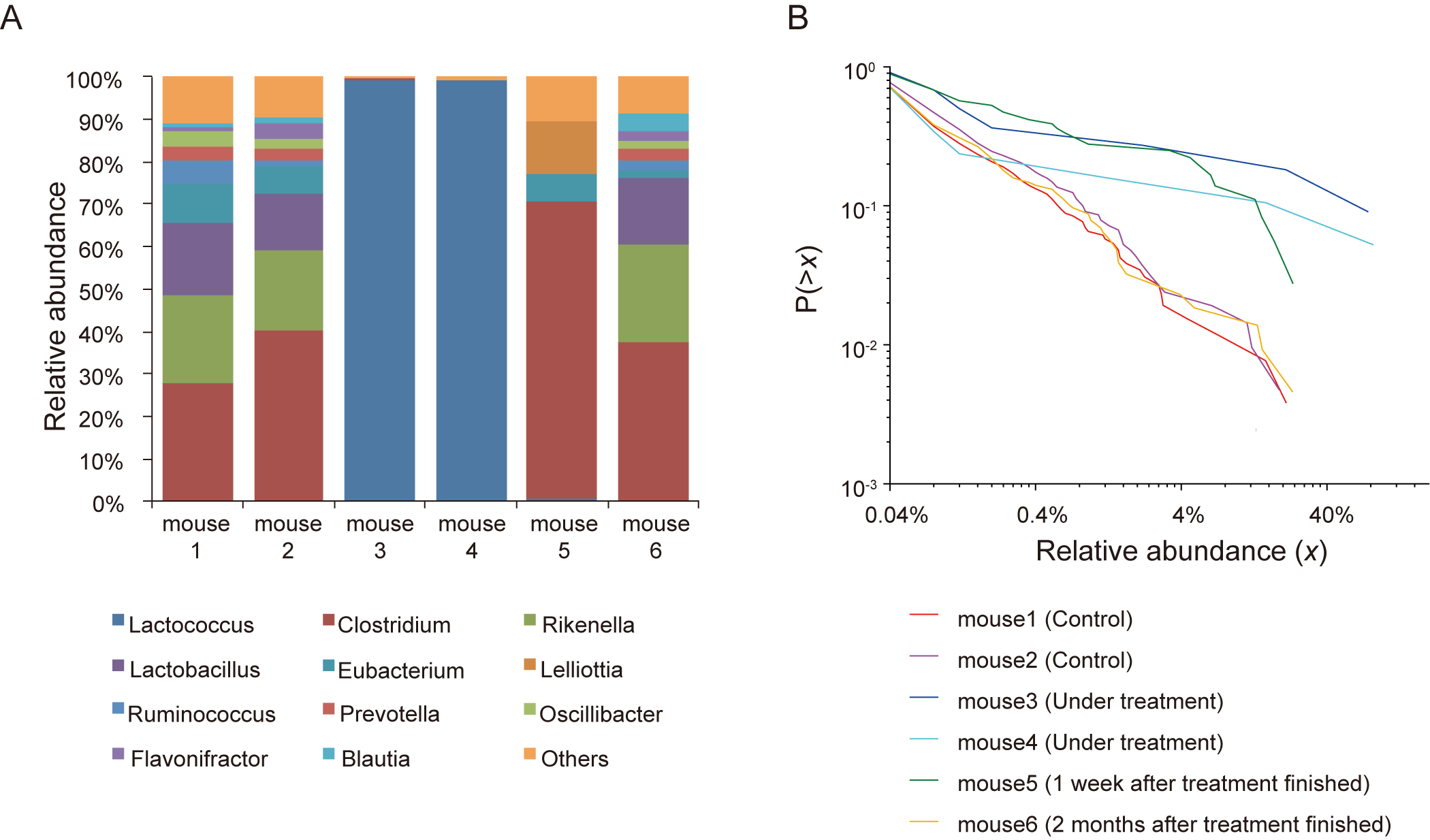

Supplement: S10 Fig — Data are from the paper by Dollive et al. [32]. Control fecal samples of two mice (mouse 1, 2), another two fecal samples of mice treated with antibiotics for 2 days (mouse 3, 4), and two fecal samples of mice in which antibiotic treatment was stopped (mouse 5, 6) were analyzed. For each sample, 2,500 high-quality 16S V1-V2 sequence data were analyzed. A: Bacterial composition at the genus level. The bacterial composition at the genus level calculated using the 16S data is shown in the bar graph. B: CRADs of antibiotic-treated mice. CRADs of the gut microbiomes of all six mice from the analysis of 16S data. (TIF) [file pone.0180863.s010.tif]

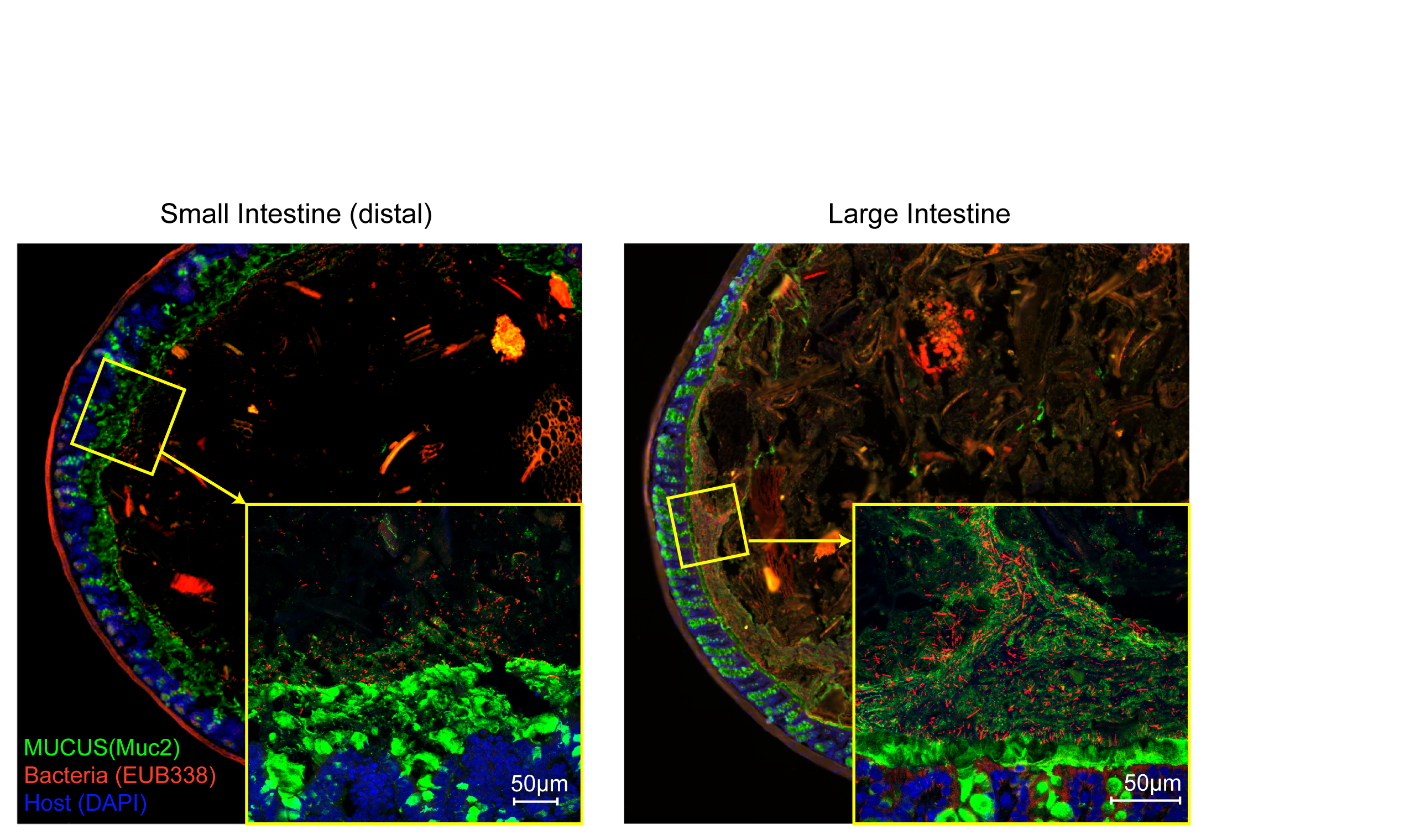

Supplement: S11 Fig — A: Visualization of the microbial distribution in the small intestine (distal). The sample was stained with Muc2 (green), EUB338 (red), and DAPI (blue). B: Visualization of the microbial distribution in the large intestine. The sample was stained with Muc2 (green), EUB338 (red), and DAPI (blue). (TIF) [file pone.0180863.s011.tif]

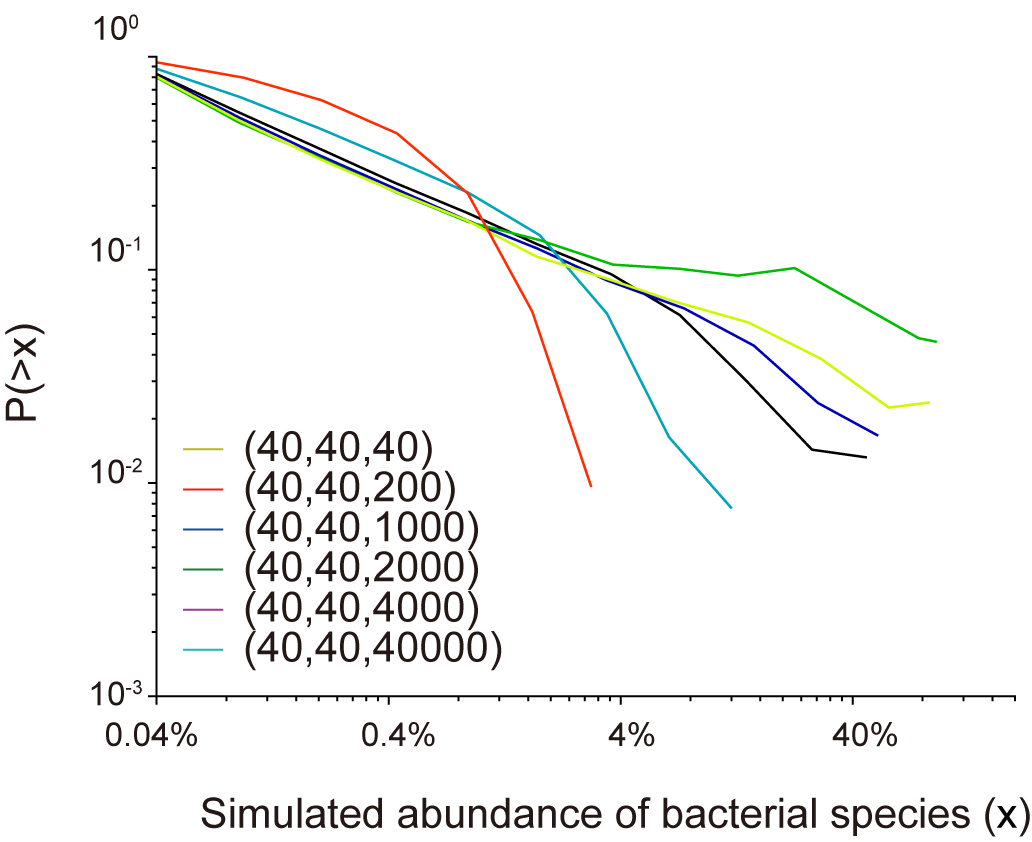

Supplement: S12 Fig — CRADs for each simulation height (40, 200, 1000, 2000, 4000, 40000) are shown. Initial bacterial cells were filled on the bottom of a 40 × 40 lattice. Initial species number was 200. Immigration probability p = 1. (TIF) [file pone.0180863.s012.tif]

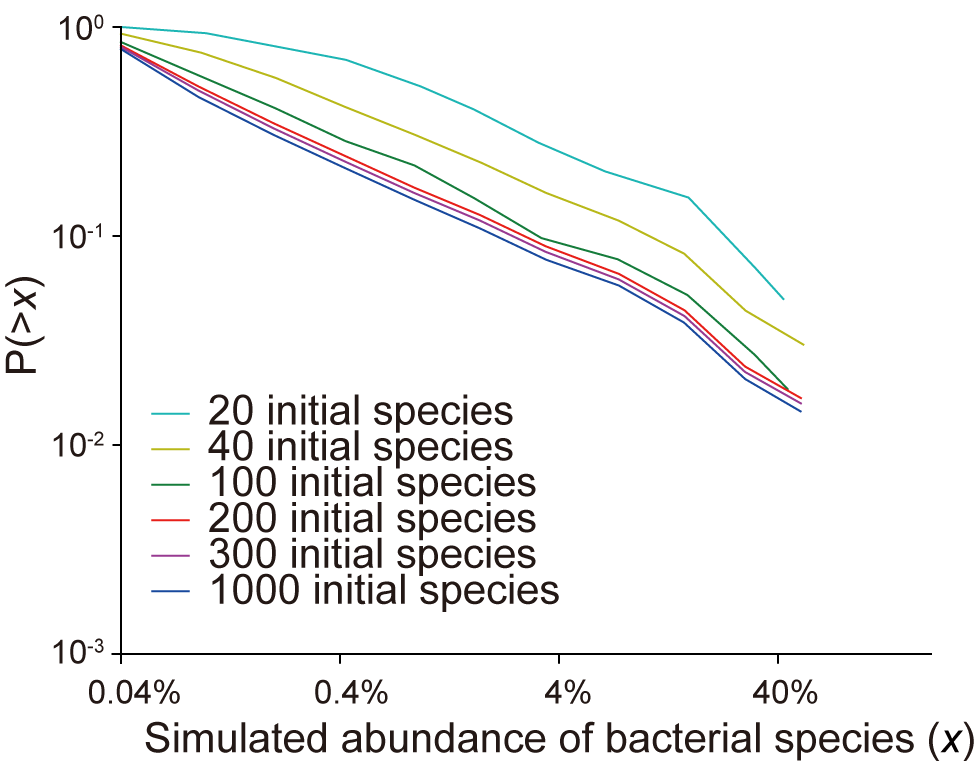

Supplement: S13 Fig — The colored solid line represents the CRADs of simulations started with different numbers of species (20, 40, 100, 200, 300, 1000). Immigration probability, p, was 1.0. Simulation size was 40 × 40 × 2000. (TIF) [file pone.0180863.s013.tif]

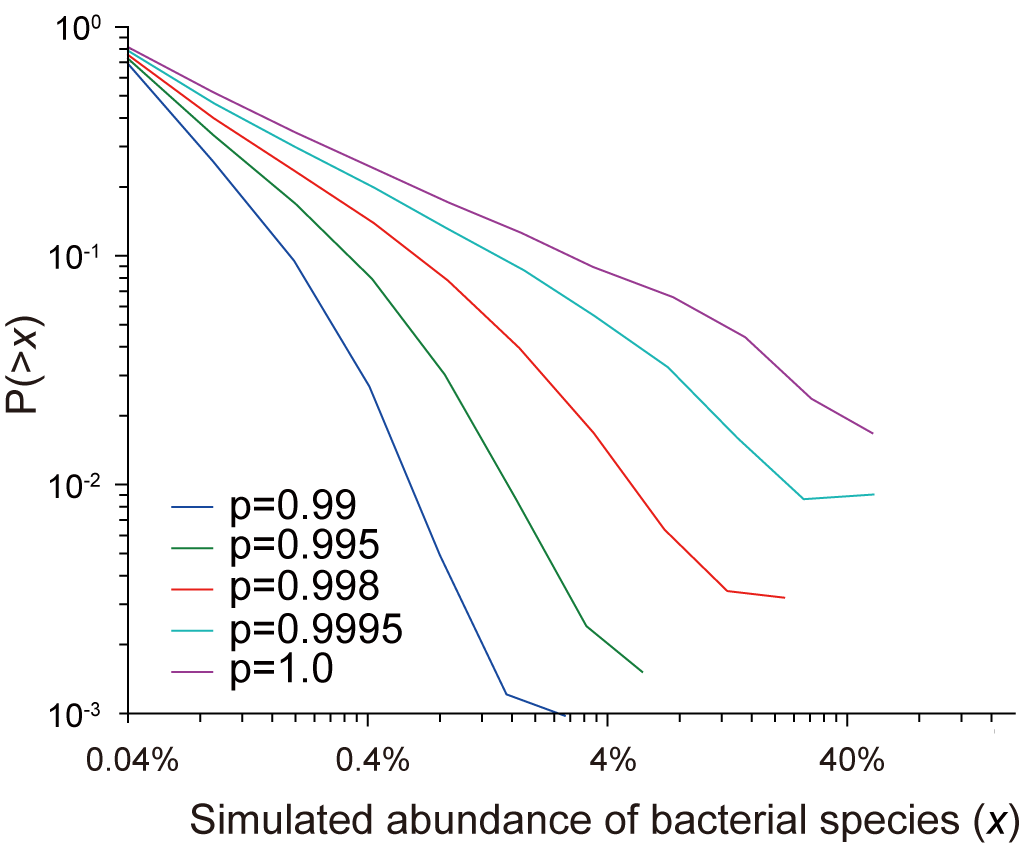

Supplement: S14 Fig — The solid line represents the CRAD of each probability p (0.99, 0.995, 0.999, 0.9995, 1.0). Simulation size was 40 × 40 × 2000. Initial species number is 200. (TIF) [file pone.0180863.s014.tif]

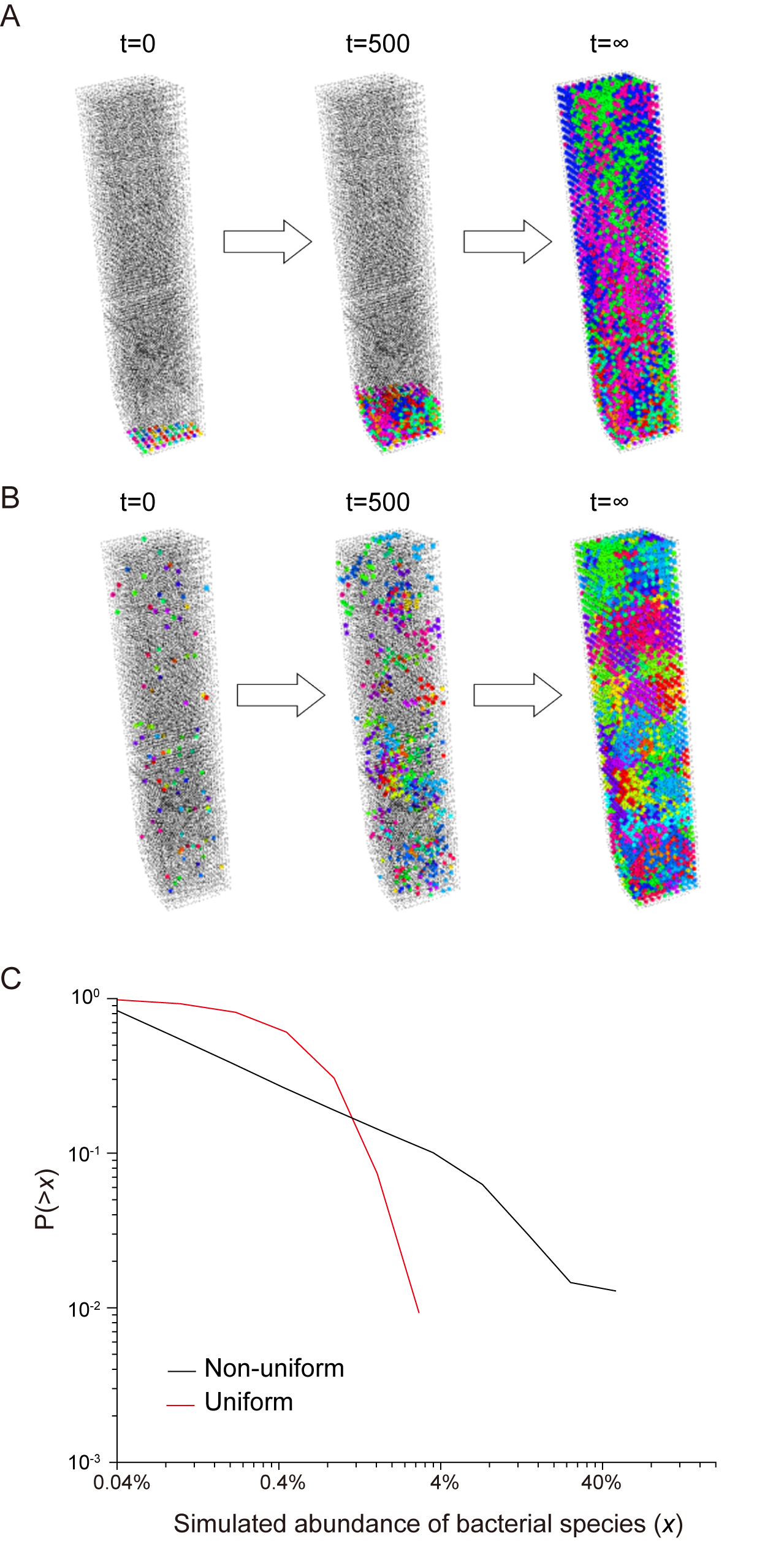

Supplement: S15 Fig — A: Image of bacterial proliferation in the simulation over time (time t = 0, 500, ∞), with non-uniform initial spatial distribution. Each color represents a different bacterial species. Simulation size was 10 × 10 × 50. Initial species number is 20. B: Image of bacterial proliferation in the simulation over time (time t = 0, 500, ∞), with a uniform initial spatial distribution. Each color represents a different bacterial species. Simulation size was 10 × 10 × 50. Initial species number is 20. C: The solid line represents the CRADs of simulations start with uniform and non-uniform spatial distributions. Simulation conditions are the same as above. (TIF) [file pone.0180863.s015.tif]
