# Supplementary material for: A 3-dimensional mathematical model of microbial proliferation that generates the characteristic cumulative relative abundance distributions in gut microbiomes
Source: PLoS One. 2017 Aug 8;12(8):e0180863. doi: 10.1371/journal.pone.0180863 (PMC5549704; doi:10.1371/journal.pone.0180863)
Supplement: S1 Appendix — (PDF) [file pone.0180863.s016.pdf]

S1 Appendix

Species richness and diversity under the power law CRADs.

Here, we mathematically derive the relation between the sampling number N and the number of observed species K, and the value of Shannon index S under the assumption of power law distribution of population x.

$$P(>x) \propto x^{-\beta} \tag{1}$$

We introduce a theoretical technique of approximation of x_i by the following deterministic series which exactly fulfills eq.(1) [1]

$$x_i = K^{\frac{1}{\beta}} i^{-\frac{1}{\beta}} \tag{2}$$

We can derive the following relation

$$\begin{aligned} N &= \sum_{i=1}^{K} x_i \\ &= \sum_{i=1}^{K} K^{\frac{1}{\beta}} i^{-\frac{1}{\beta}} \\ &\approx K^{\frac{1}{\beta}}[1 + \frac{\beta}{\beta-1}((K+1)^{-\frac{1}{\beta}+1} - 2^{-\frac{1}{\beta}+1})] \end{aligned} \tag{3}$$

where the summation is approximated by the following integral.

$$\begin{aligned} \sum_{i=1}^{K} i^{-\frac{1}{\beta}} &\approx 1 + \int_{2}^{K+1} i^{-\frac{1}{\beta}} di \\ &= 1 + \frac{\beta}{\beta-1}((K+1)^{-\frac{1}{\beta}+1} - 2^{-\frac{1}{\beta}+1}) \end{aligned} \tag{4}$$

Apparently, eq.(3) looks singular at $\beta = 1$, however, it is a continuous function of β, actually in the limit of $\beta \to 1$,

$$\lim_{\beta \to 1} N \approx K[1 + \log{(K+1)} - \log 2] \tag{5}$$

Shannon index S is calculated in the same way as follows:

$$\begin{aligned} S &\equiv -\sum_{i=1}^{K} \frac{x_i}{N} \log \frac{x_i}{N} \\ &= -\sum_{i=1}^{K} \frac{K^{\frac{1}{\beta}} i^{-\frac{1}{\beta}}}{N} \log \frac{K^{\frac{1}{\beta}} i^{-\frac{1}{\beta}}}{N} \\ &\approx -\frac{K^{\frac{1}{\beta}}}{N} \log \frac{K^{\frac{1}{\beta}}}{N} - \int_{2}^{K+1} \frac{K^{\frac{1}{\beta}} i^{-\frac{1}{\beta}}}{N} \log \frac{K^{\frac{1}{\beta}} i^{-\frac{1}{\beta}}}{N} di \\ &= -\log \frac{K^{\frac{1}{\beta}}}{N} - \frac{1}{\beta-1}[1 - \frac{K^{\frac{1}{\beta}}}{N}\{1 + \frac{\log{(K+1)}}{(K+1)^{\frac{1}{\beta}-1}} - \frac{\log 2}{2^{\frac{1}{\beta}-1}}\}] \end{aligned} \tag{6}$$

Again, it looks singular at $\beta = 1$, it is a continuous function of β. In the limit of $\beta \to 1$,

$$\lim_{\beta \to 1} S \approx \log \frac{N}{K} + \frac{K}{N}[(\log{(K+1)})^2 - (\log 2)^2] \tag{7}$$

References

1. Takayasu M, Watanabe H, Takayasu H. Generalised Central Limit Theorems for Growth Rate Distribution of Complex Systems. J Stat Phys 2014 Feb;155:47-71
